# Building School Behavioral Health Capacity: A Scoping Review of Evidence-Based Ingredients Delivered by Paraprofessionals

**DOI:** 10.3390/bs16060835

**Published:** 2026-05-22

**Authors:** Bailey R. Dow, Savannah B. Simpson, Samuel D. McQuillin, Dodie Limberg, Kimberly J. Hills, Eugene S. Huebner

**Affiliations:** 1 Department of Psychology, University of South Carolina, Columbia, SC 29208, USA; 2 Department of Educational and Developmental Science, University of South Carolina, Columbia, SC 29208, USA

**Keywords:** paraprofessionals, school behavioral health, social-emotional support, evidence-based ingredients, task-shifting

## Abstract

Youth are increasingly struggling with mental health, yet many lack access to formal care. Evidence indicates that building coping skills can improve mental health and wellbeing. School personnel may be well-positioned to help youth build these coping skills by delivering discrete evidence-based ingredients in their everyday interactions and relationships with students. This scoping review synthesizes the literature on social-emotional evidence-based ingredients delivered by paraprofessionals and explores their potential application in school behavioral health. We searched PsycINFO and PubMed, screened 200 titles/abstracts and 46 full-texts, and yielded 19 studies from which we synthesized data using the RE-AIM framework. We identified 17 evidence-based ingredients, with the most common being mindfulness, relaxation, psychoeducation, exposure, and cognitive restructuring. These were delivered in various formats and settings by different paraprofessionals (e.g., graduate students, teachers, caregivers), with most paraprofessionals receiving some training and supervision. Thirteen studies showed significant improvements in at least one outcome (i.e., anxiety, depression, suicidality, wellbeing). Six studies examined long-term effects, with mixed findings. Despite variation in delivery and training, paraprofessionals appear to feasibly and effectively deliver evidence-based ingredients. These findings support task-shifting ingredients as a scalable approach for supporting youth mental health within school behavioral health systems.

## 1. Introduction

Today’s youth face unprecedented levels of anxiety, depression, and suicidality (Centers for Disease Control and Prevention [CDCP], 2023; Cigna, 2018; McGorry et al., 2025; Twenge, 2023), yet more than half of them do not receive the mental health care they need (Barican et al., 2022; Koppelman, 2004). Evidence indicates that increasing coping skills in young people is one way to increase wellbeing and reduce impairment associated with these and other disorders (Frydenberg et al., 2021; Ronen, 2021). In conceptualizing youth mental health, it is important to consider both the presence of psychological distress and the presence of wellbeing. The dual factor model of mental health posits that mental health is not simply the absence of symptoms but also includes positive indicators such as life satisfaction and emotional wellbeing (Suldo & Shaffer, 2008). This framework highlights the importance of both reducing distress (e.g., anxiety, depression, suicidality) and promoting strengths (e.g., coping, resilience).

Schools are a primary setting for reaching large numbers of youth and are increasingly tasked with delivering comprehensive mental and behavioral health services (Hoover & Bostic, 2021; Panchal et al., 2025; Shahidullah, 2019). Frameworks such as multi-tiered systems of support (MTSS), positive behavior intervention and support (PBIS), and response to intervention (RTI) emphasize the importance of providing a continuum of behavioral health services within schools (Finch et al., 2015; McIntosh & Goodman, 2016; Sugai & Horner, 2009). For effective implementation of these frameworks, schools must select evidence-based psychosocial interventions and focus on building workforce capacity and providing adequate implementation supports (Kittelman et al., 2021; Mahoney, 2020; Rowe et al., 2021; Stoiber & Gettinger, 2015; Ward et al., 2021). Lay helpers have the potential to foster coping skills and resilience in youth through accessible, community-based support. Equipping trusted community members with evidence-based strategies improves youth wellbeing, helps address the shortage of mental health professionals (e.g., Butryn et al., 2017; National Center for Health Workforce Analysis, 2024), and reduces barriers to accessing care (e.g., Anderson et al., 2017). Within school behavioral health systems, a wide range of non-licensed individuals, such as teachers, teaching assistants, peers, caregivers, mentors, and community lay workers, could play a role in the delivery of psychosocial supports. It is critical to understand which discrete psychosocial practices can be feasibly delivered by these individuals and under what implementation conditions, to strengthen school-based behavioral health systems and amplify positive student and school outcomes. With appropriate guidance and supervision, non-licensed adults may be able to extend the reach of school behavioral health services by delivering discrete skills from evidence-based psychosocial interventions—referred to here as evidence-based ingredients.

For the purposes of this review, we distinguish between evidence-based psychosocial interventions (EBPIs) and the discrete components within them. EBPIs refer to manualized or protocolized interventions that have demonstrated efficacy in improving mental health outcomes (e.g., Lyon et al., 2020). Within these interventions are evidence-based ingredients, defined here as specific, theory-driven skills or practices that target empirically supported mechanisms of change (e.g., cognitive restructuring to modify maladaptive beliefs, behavioral activation to increase engagement in reinforcing activities). This distinction is important because terminology in the literature is not always used consistently; in some cases, “evidence-based practices” has been used to refer to discrete techniques or strategies rather than full intervention packages. To reduce ambiguity, we use the term EBPIs to refer to comprehensive intervention models and evidence-based ingredients to refer to their active components. For example, a cognitive behavioral therapy protocol would be considered an EBPI, whereas cognitive restructuring or exposure would represent evidence-based ingredients within that intervention. Although EBPIs are typically delivered as multi-session packages, research suggests that practitioners often implement only select components in practice (Becker et al., 2013). Accordingly, the current review focuses on these discrete ingredients as the unit of analysis, given their potential for flexible delivery and task-shifting.

Additionally, the term paraprofessional refers to any individual without a formal clinical license who delivers structured psychosocial support to youth (e.g., lay counselors, mentors, teachers, teaching assistants, coaches). This definition is intentionally broad and reflects how schools operationalize behavioral health service delivery within MTSS frameworks, where multiple workforce roles contribute to tiered intervention delivery under varying levels of supervision. In this review, paraprofessional-delivered ingredients are examined as implementation-relevant exemplars, or instances in which discrete psychosocial ingredients were delivered by non-licensed individuals in school or community contexts, allowing for examination of training, supervision, and implementation supports associated with successful delivery.

### 1.1. Evidence-Based Psychosocial Interventions

In the field of mental health, the term “evidence-based practices” (EBPs) has been described as prevention and intervention strategies that improve client outcomes supported by the integration of the best available research evidence, clinical expertise and judgement, and the context of the client (i.e., characteristics, culture, values) (American Psychological Association Presidential Task Force on Evidence-Based Practice, 2006). To reduce ambiguity in terminology, we use the term evidence-based psychosocial interventions (EBPIs) to refer to manualized or protocolized interventions that have demonstrated efficacy in improving mental health outcomes.

Extensive research in medicine and clinical psychology supports the effectiveness of EBPIs in improving healthcare systems and treating physical and mental health challenges, often demonstrating greater outcomes compared to usual care (Connor et al., 2023; Melnyk & Fineout-Overholt, 2022; J. R. Weisz et al., 2013; J. R. Weisz et al., 2017). For decades, psychologists have considered these interventions as the ‘gold standard’ for mental health care as they promote research-driven decision making, reduce variations in practice, and improve patient outcomes (American Psychological Association Presidential Task Force on Evidence-Based Practice, 2006; Connor et al., 2023). However, mental health professionals also face many challenges in implementing EBPIs, such as the financial resources to access them, the training and time to learn them, and the lack of flexibility to adapt them to their individual client needs (Frank et al., 2020; Kazdin, 2019; Lilienfeld et al., 2013; Peters-Corbett et al., 2024; Stirman et al., 2016). As a result, less than ten percent of clinicians fully implement manualized intervention protocols with their clients, with most incorporating only select components (Becker et al., 2013). These challenges highlight the gap between research and practice and underscore the need for more adaptable and accessible approaches to effectively support youth mental health.

#### Modular and Transdiagnostic Approaches

Researchers have therefore explored alternative approaches that retain the core elements of EBPIs while allowing for greater flexibility. Wampold and Imel’s (2015) *Contextual Model* conceptualizes these core elements as common factors (i.e., relationship qualities, expectations about self and treatment) and specific ingredients (e.g., relaxation techniques, problem-solving, exposure) that together serve as the mechanisms of change for clinical intervention. Subsequent research has supported the integration of common factors and specific ingredients for effective treatment (e.g., Wampold & Ulvenes, 2019). As a reminder, the term ‘evidence-based ingredients’ is used here to encompass these specific ingredients that are rooted in EBPIs and harness the mechanism(s) of change for the desired outcomes.

Modular treatments emerged as promising ways for brief, flexible treatment that leverages evidence-based ingredients. Modular treatments distill manualized interventions into discrete activities and skills (i.e., evidence-based ingredients) that can be flexibly applied to achieve desired outcomes (Chorpita et al., 2005). Examples of modular treatments include the Modular Approach to Therapy for Children with Anxiety, Depression, Trauma, or Conduct Problems (MATCH; Chorpita & Weisz, 2009).

Relatedly, transdiagnostic approaches, such as FIRST principle-guided psychotherapy (J. R. Weisz & Bearman, 2020; J. Weisz et al., 2017), emphasize empirically supported principles of change that cut across diagnostic categories (e.g., feeling calm, increasing motivation, changing thoughts, solving problems), rather than focusing solely on discrete components. Although both modular and transdiagnostic approaches promote flexibility, modular treatments primarily focus on selecting and sequencing discrete evidence-based ingredients, whereas transdiagnostic approaches emphasize broader processes that organize and guide the use of these components. These principles are often operationalized through the delivery of discrete practices, making transdiagnostic approaches compatible with an ingredient-focused framework.

Modular treatments offer independent evidence-based ingredients that can be used to meet the evolving needs of young people. Researchers have seen positive clinical outcomes as a result of modular treatments, such as decreased internalizing and externalizing problems in pre- to post-treatment comparisons (J. Weisz et al., 2017; J. R. Weisz & Bearman, 2020), and when compared to usual care or standard manualized interventions (Chorpita et al., 2013; Chorpita et al., 2017; J. R. Weisz et al., 2012, 2013). Researchers have found that modular treatments designed to be delivered within a single session are effective in reducing mental health symptoms, with only marginally smaller effect sizes than those of lengthier, multi-session interventions (Schleider & Weisz, 2017; J. R. Weisz et al., 2017). These findings suggest that shorter, flexible interventions can yield significant benefits, offering promising alternatives to traditional, long-term mental health care.

From an implementation perspective, modular approaches align well with the structures of school behavioral health systems, namely MTSS. MTSS is a data-driven, tiered framework designed to proactively identify and address students’ social, emotional, behavioral, and academic needs through interventions of increasing intensity (McIntosh & Goodman, 2016). Within this model, students receive supports that range from universal, preventative efforts (Tier 1) to targeted group interventions (Tier 2) to intensive, individualized services (Tier 3), and they can receive multiple tiers of support at once (Brown-Chidsey & Wilkinson, 2025; Kilgus & von der Embse, 2019; Marsh & Mathur, 2020). In addition to this tiered support system, key features of MTSS include universal screening, ongoing progress monitoring, multidisciplinary team meetings, and data-based decision-making (Brown-Chidsey & Wilkinson, 2025). These features create a need for interventions that are adaptable in intensity, format, and duration to be flexibly applied across tiers. Modular interventions may be particularly well suited to this context, as their discrete, evidence-based ingredients can be selected and sequenced in response to student needs and responsiveness to intervention. This flexibility supports implementation across a range of service formats (e.g., brief individual sessions, small groups, classroom-based support) and aligns with the iterative, data-informed nature of MTSS. Additionally, school behavioral health services are delivered by individuals with diverse roles and levels of expertise, such as teachers, school psychologists, counselors, school mental health providers, interventionists, behavioral specialists, nurses, peers, and other school personnel (Arora et al., 2019; Loftus-Rattan et al., 2023; Radley & Dart, 2019), highlighting the importance of interventions that are adaptable and feasible across providers. Modular approaches may be well-positioned to accommodate this variability by offering structured yet flexible components that can be implemented by individuals with differing training and experience. Recent extensions of MTSS, namely the Interconnected Systems Framework (ISF), further emphasize the integration of school mental health and behavioral supports within a coordinated, team-based model, increasing the need for interventions that can be flexibly implemented across systems and providers (S. Barrett et al., 2013; Eber et al., 2019; Weist et al., 2022). Overall, modular approaches may offer a promising fit for the integrated, team-based structure emphasized by MTSS and ISF and the logistical and organizational demands of school-based service delivery.

### 1.2. Task-Shifting and Implementation Support in School Behavioral Health Systems

Task-shifting is the process of delegating and redistributing certain tasks from highly trained professionals to those with less training or fewer credentials, with guided supervision and support, so that the reach and quality of services are improved (McInnis & Merajver, 2011; Patel et al., 2007; World Health Organization, 2008). Evidence suggests that evidence-based ingredients can be task-shifted to paraprofessionals (e.g., peers, counselors, lay workers) with great success (Barbui et al., 2020; Bolton et al., 2014; Depping et al., 2021; Patel et al., 2017; Singla et al., 2021; Taha et al., 2026). For example, Patel et al. (2017) studied the feasibility of lay counselors delivering brief interventions for those struggling with depression in primary care in India and found that the inclusion of a brief intervention (i.e., Healthy Activity Program—evidence-based ingredient of behavioral activation) improved symptoms compared to enhanced usual care (i.e., usual care with a depressive symptom screener and psychiatric care referrals). These improvements appeared to be relatively stable over time, as those who received the brief intervention from lay counselors maintained their improvements over the following year, mediated by their behavior activation at three months after treatment (Weobong et al., 2017).

Much of this research has focused on supporting adults; however, the same evidence-based ingredient task-shifting efforts can be extended to youth as well (Hill et al., 2025). Mental health professionals who work with youth can task-shift teaching and supporting coping skills to less specialized paraprofessionals. Indeed, mental health professionals have begun task-shifting targeted interventions, counseling, and academic support services to mentors (S. McQuillin et al., 2011, 2015; S. D. McQuillin & McDaniel, 2020). For instance, S. D. McQuillin and McDaniel (2020) conducted a pilot study where mentors were task-shifted to deliver a goal-focused intervention for elevated disruptive behaviors. Mentors felt that delivering the intervention was feasible, and mentees experienced lower behavior infractions, school problems, and emotional problems (S. D. McQuillin & McDaniel, 2020). However, these extant studies have primarily focused on multi-session curricula (i.e., 10+ sessions in the S. D. McQuillin and McDaniel (2020) study), and less attention has been placed on the discrete tasks or ‘ingredients’. Although promising research exists on the utility of paraprofessional delivery of evidence-based ingredients for adult populations, few researchers to date have examined paraprofessional-delivered evidence-based ingredients for youth or how they can be delivered through task-shifting.

In school contexts, task-shifting must be embedded within pre-existing support structures, such as MTSS. Training alone is often insufficient; coaching, consultation, and ongoing supervision are critical for ensuring fidelity, safety, and effectiveness, especially when delivering practices that target mental health challenges. Despite the importance of these supports, relatively little work has examined how evidence-based ingredients have been implemented by paraprofessionals in school or community settings, or what implementation supports accompanied their delivery. Although several systematic and scoping reviews have examined paraprofessional-delivered interventions (e.g., Singla et al., 2021) or youth mental health interventions (e.g., J. R. Weisz et al., 2017) more broadly, these reviews have typically focused on program-level effectiveness, general intervention outcomes, and multi-session intervention programs, leaving important gaps in understanding how discrete evidence-based ingredients are implemented in practice. The current scoping review addresses this gap by mapping the literature on paraprofessional-delivered evidence-based ingredients for youth and evaluating their potential for integration within school behavioral health systems and multi-tiered frameworks. Scoping reviews are well-suited for addressing such questions, as they are designed to comprehensively chart the state of the literature, identify key concepts, clarify definitions, and pinpoint gaps in research (Arksey & O’Malley, 2005). In contrast to systematic reviews, scoping reviews explore the breadth of evidence, capture heterogeneous study designs, and summarize how interventions, or ingredients, have been implemented across contexts rather than assessing their effectiveness alone (Munn et al., 2018). Accordingly, a scoping review provides an appropriate methodological framework for synthesizing implementation-relevant evidence on paraprofessional-delivered ingredients and informing future efforts to scale these practices within school behavioral health.

### 1.3. Purpose of Current Review

The purpose of this review is to synthesize the literature on social-emotional evidence-based ingredients that have been delivered by paraprofessionals to youth in school and community contexts. Specifically, we aimed to (a) identify the discrete evidence-based ingredients that have been delivered, (b) describe the workforce roles involved in delivery, and (c) examine the training, supervision, and implementation supports reported across studies. By centering discrete activities and implementation features rather than program packages, this review seeks to inform task-shifting within school behavioral health and the training and supervision structures that support paraprofessionals in delivering evidence-based ingredients. Given the breadth of social-emotional challenges, the current review focused on social-emotional evidence-based ingredients specific to the needs of today’s youth. The National Mentoring Partnership identified that youth currently struggle with anxiety, depression, and suicidality at two to four times the rate of prior generations (Garringer & Benning, 2023). Consistent with the dual factor model of mental health introduced above, and aligned with the Positive Youth Development perspective, this framework emphasizes considering youth wellbeing in addition to mental health difficulties (Suldo & Shaffer, 2008). Most commonly, this is captured via subjective wellbeing, which is defined as the combination of life satisfaction and positive affect relative to negative affect (Diener, 2000; Suldo & Shaffer, 2008). Evidence from the field of positive psychology suggests that resilience, gratitude, hope, and self-compassion are strongly associated with subjective wellbeing and overall life satisfaction in youth (e.g., Bag et al., 2022; Kardas et al., 2019; Pleeging et al., 2021). Therefore, in the context of this review, we included these related positive psychology constructs as part of subjective wellbeing to capture interventions aimed at promoting subjective wellbeing. This review also captures the reduction in distress, including indicators of ill-being such as anxiety, depression, and suicidality. Overall, the current review focused on social-emotional evidence-based ingredients that address youth anxiety, depression, suicidality, and subjective wellbeing.

## 2. Method

### 2.1. Eligibility Criteria

To be eligible for inclusion in the scoping review, all studies met criteria related to language, participant demographics, study design, and variables of interest. Overall, the studies included were original, peer-reviewed articles published in or translated into English that investigated social-emotional evidence-based practices or ingredients delivered by paraprofessionals to youth under 18 years of age. All types of research designs were included (i.e., quantitative, qualitative, mixed-methods studies). See Table 1 for a thorough description of the inclusionary and exclusionary criteria applied to determine eligibility for analysis.

For this review, paraprofessionals were defined as individuals who do not hold a formal mental health license. This includes individuals with partial professional training, such as graduate students, school nurses, or teachers, who may provide services or certain interventions as part of their role but are not independently licensed mental health providers. Additionally, we included recipients of the intervention who self-administered the intervention via technology (e.g., app, website) as paraprofessionals, given that those recipients were not licensed mental health professionals. This definition of paraprofessionals was applied consistently during study selection to ensure that included interventions were delivered by non-licensed personnel while acknowledging variability in training backgrounds.

Additionally, for this review, evidence-based ingredients were defined as discrete, theory-informed practices or skills that have empirical support as mechanisms of change in evidence-based psychosocial interventions (i.e., full interventions) targeting youth mental health or social-emotional development (e.g., exposure, cognitive restructuring, relaxation, problem solving, goal setting, behavioral activation, emotion labeling). Structured activities were only considered evidence-based ingredients if they could be linked to established evidence-based mechanisms rather than being classified solely on the basis of structure or intentionality. Identification of these ingredients and the search terms used were guided by prior literature on common practice elements (e.g., Chorpita et al., 2005; Chorpita & Weisz, 2009; J. R. Weisz & Bearman, 2020) and how each study described the targeted skills, processes, or mechanisms addressed in the intervention. This review did not empirically test whether each ingredient independently demonstrated evidence-based status; rather, ingredients were identified as evidence-based if they had prior empirical support as mechanisms of change in the broader youth mental health and modular treatment literature (e.g., Fitzpatrick et al., 2023; J. R. Weisz et al., 2012).

This scoping review was informed by the Joanna Briggs Institute (JBI) methodological guidance for scoping reviews (Peters et al., 2020) and is reported in alignment with the PRISMA extension for scoping reviews (PRISMA-ScR; Tricco et al., 2018). This review was not registered in PROSPERO. This project was completed as part of a student’s comprehensive examination; thus, the review process was conducted by a single coder. Screening and eligibility decisions were conducted by the first author using predefined inclusion criteria applied consistently across all stages. To ensure transparency and trustworthiness within this constraint, all coding decisions were documented, reviewed against established scoping review rubrics, and externally evaluated by faculty raters who reached unanimous agreement that the project met standards for comprehensiveness and rigor.

### 2.2. Search Strategies and Results

The search strategy was developed by the author with the collaboration of a research librarian. Articles for the review were identified through searches in the electronic databases PsycINFO and PubMed, which were selected to prioritize peer-reviewed research on mental health, social-emotional interventions, and paraprofessional delivery. Although additional databases may index relevant studies, the search strategy emphasized sources most likely to capture research on evidence-based ingredients and paraprofessional-delivered interventions within behavioral health contexts. We acknowledge this tradeoff and note that some relevant studies outside these two databases may have been omitted. For example, relevant studies indexed in broader interdisciplinary databases (e.g., Web of Science, ERIC, CINAHL) may not have been captured. Additionally, we did not conduct searches of grey literature, hand-search reference lists, or perform forward/backward citation tracking, as this would have broadened the review beyond its stated aims and intended scope. We note this as a limitation of the current review. Accordingly, the findings reflect peer-reviewed studies of paraprofessional-delivered social-emotional evidence-based ingredients identified within the selected databases.

Searches in PsycINFO and PubMed were conducted using Boolean operators (“OR,” “AND”) to combine conceptually related sets of search terms representing five key domains: (a) youth populations, (b) target outcomes measured (i.e., anxiety, depression, suicidality, subjective wellbeing, life satisfaction, positive affect), (c) evidence-based social-emotional skills (i.e., using terms from common practice elements identified in Chorpita et al., 2005; Chorpita & Weisz, 2009; and J. R. Weisz & Bearman, 2020, as these have a demonstrated evidence-base to support them as mechanisms of change), (d) paraprofessional or lay provider roles, and (e) the discrete ingredients. It is important to note that suicidality was included as a targeted outcome given its clinical and public health importance (Kalin, 2021). Suicidal ideation and behaviors may occur independently of depressive symptoms and often require targeted prevention and intervention approaches (Calear et al., 2016; Rhodes & Bethell, 2008).

Proximity searching and the wildcard asterisk (*) were frequently used to maximize search results. The search histories were documented in Table 2 using database-generated notation (e.g., S1, S2), with each numbered search representing a distinct set of terms. Specifically, S1 captured terms related to youth (e.g., “youth*,” “adolescen*,” “student*”), S2 captured terms related to targeted mental health and wellbeing outcomes (e.g., “anxiety,” “depression,” “subjective wellbeing”), S3 captured terms related to evidence-based social-emotional skills (e.g., “cognitive restructur*,” “behavioral activation,” “social-emotional,” “self-management”), S4 captured terms related to paraprofessionals and lay providers (e.g., “mentor*,” “lay*,” “paraprofessional*,” “coach*”), and S5 captured terms related to the brief, discrete ingredients (e.g., “brief,” “single session intervention,” “module,” “ingredient”). These terms were then combined using “AND” to yield the final results. This multi-search approach allowed systematic refinement and transparent reporting of how each conceptual domain contributed to the overall search strategy. The literature search was conducted from database inception through 20 January 2025, with no date restrictions applied to ensure comprehensive coverage of relevant studies.

**Figure 1:**
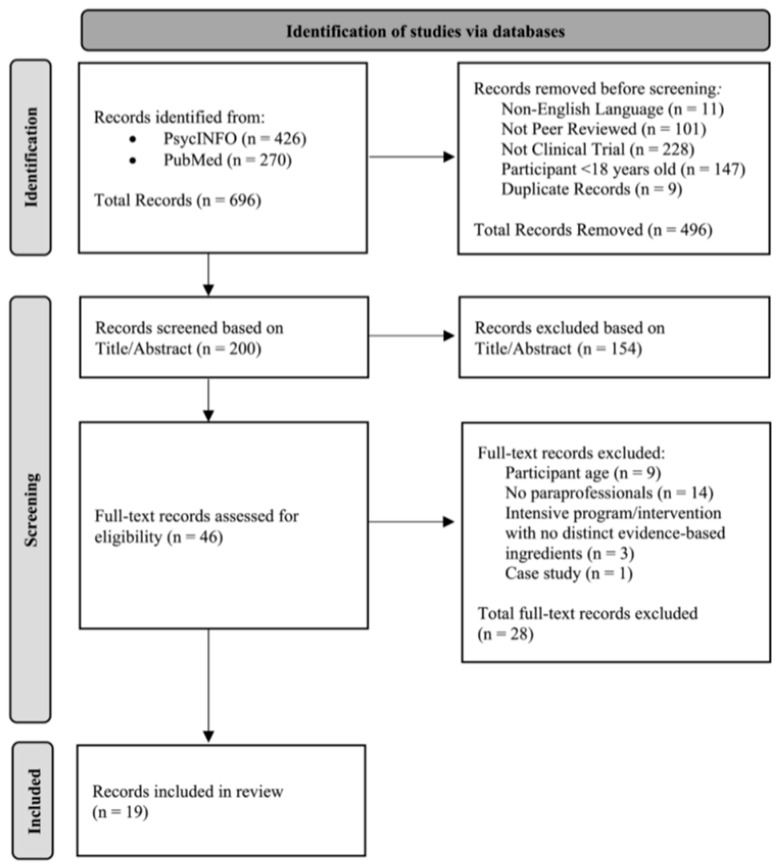
PRISMA Flow Diagram. *Note*. Retrieved From: Page et al. (2021).

The search procedure described above (and in Table 1 and Table 2) yielded 200 records, excluding studies not published in or translated into English, studies that had not been peer-reviewed, and duplicate studies. Screening and data management were conducted using standard spreadsheet tools rather than dedicated systematic review software (e.g., Rayyan, Covidence). The author screened the titles and abstracts of the 200 records to determine if the articles met the inclusionary criteria detailed in Table 1, ensuring relevance to the scoping review at face value. After screening the titles and abstracts, a full-text review was conducted on 46 records to assess eligibility, which included any questionable articles. The full-text review resulted in 19 studies that met the eligibility criteria, and these studies were included in the current scoping review. See Figure 1 for the PRISMA flow diagram (Page et al., 2021; Tricco et al., 2018) that depicts the process for selecting records to include in the current scoping review.

### 2.3. Article Coding

The 19 records eligible for inclusion in the scoping review were read to extract the following information from each article: (1) study design, (2) sample size, (3) sample demographics (e.g., age, gender/sex, race/ethnicity, location), (4) outcome(s) evaluated (i.e., anxiety, depression, suicidality, subjective wellbeing), (5) intervention, (6) evidence-based ingredient, (7) results, (8) delivery information (i.e., paraprofessional, setting, duration), and (9) training information (i.e., from whom, duration, resources). See Table A1 and Table A2 for detailed information extracted from each study. Consistent with scoping review methodology, formal quality appraisal of included studies was not conducted, as the purpose of this review was to map the scope and characteristics of the existing literature.

### 2.4. Synthesized Results

After charting the data from the 19 included records, the data were synthesized and organized based on the RE-AIM framework (Glasgow et al., 1999; Holtrop et al., 2021) to consider the reach, effectiveness, adoption, implementation, and maintenance of social-emotional evidence-based ingredients delivered by paraprofessionals. The objectives of inquiry were: (a) Reach—the identification of who has received paraprofessional-delivered social-emotional evidence-based ingredients, (b) Effectiveness—the impact (i.e., youth outcomes) of social-emotional evidence-based ingredients that have been delivered by paraprofessionals, (c) Adoption—the identification of which paraprofessionals have delivered social-emotional evidence-based ingredients and the settings of delivery, (d) Implementation—the cost (i.e., resources, time to deliver, training) of paraprofessional-delivered social-emotional evidence-based ingredients, and (e) Maintenance—the long-term impact of paraprofessional-delivered social-emotional evidence-based ingredients. The RE-AIM framework was applied as an organizing structure for synthesizing findings across studies, rather than as a criterion for study inclusion or as a measure of whether studies explicitly reported RE-AIM domains. That is, data extracted from each study were interpreted and grouped according to the conceptual dimensions of Reach, Effectiveness, Adoption, Implementation, and Maintenance, to facilitate a comprehensive understanding of where and how paraprofessionals have delivered social-emotional evidence-based ingredients.

## 3. Results

### 3.1. Descriptive Information

The 19 studies in the current scoping review were published across a 46-year timespan, from the years 1978 to 2024. Over half of the studies (*n* = 11, 57.89%) were published more than five years ago (i.e., 1978 to 2013), and eight (42.11%) were published within the last five years, from 2020 onwards. Nearly all the studies in this review employed quantitative designs (*n* = 17, 89.47%), with only one study employing a qualitative design (Fleming et al., 2016). One ‘proof-of-concept’ study encompassed a series of studies that used both quantitative and qualitative designs (Swierad & Williams, 2023). Specifically, the quantitative designs employed across studies were experimental (*n* = 8; randomized controlled trials *n* = 6, cluster randomized trial *n* = 1, factorial design *n* = 1), quasi-experimental (*n* = 8), and observational (*n* = 1), with six of these being pilot studies and one being a ‘proof-of-concept’ study. More than two-thirds of these studies were conducted in the U.S. (*n* = 13, 68.42%) across various states in the Northeast, Midwest, and South. The remaining studies were conducted across five other countries: Australia (*n* = 2, 10.53%), Indonesia (*n* = 1, 5.26%), New Zealand (*n* = 1, 5.26%), Singapore (*n* = 1, 5.26%), and Sweden (*n* = 1, 5.26%). Study characteristics with citations are detailed in Appendix A Table A1.

### 3.2. Reach

Across the 19 studies included, this scoping review analyzed data from 4321 participants. Participant characteristics and citations are summarized in Appendix A Table A1. Sample sizes ranged from eight participants (Jennings & Jennings, 2013) to 2130 participants (Volungis, 2020), with an average sample size of approximately 227.42 participants (*SD* = 481.44). All participants were children and adolescents between four and 18 years old (*M* = 11.82, *SD* = 1.24). Participants’ gender and/or sex were reported by 15 studies (78.95%), providing percentages of females (*M* = 54.25%) and males (*M* = 45.76%) in their samples. Only one study (Kresovich et al., 2023) provided an additional gender option (i.e., “another gender identity”); however, those participants were not included in their final sample due to attrition and the low proportion for comparison analyses. Six of these studies reported approximately equal percentages of males and females. The other nine studies had one gender encompassing over 60% of the sample, with three studies reporting more males than females and six reporting more females than males.

Participants’ race and ethnicity were reported in 10 studies (52.63%), with most (*n* = 8) of those studies reporting the race and ethnicity of their specific sample and two of those studies reporting the race and ethnicity of the school from which their sample was taken. Within those 10 studies, eight were conducted within the U.S., with the race and ethnicity options including White, Black or African American, Asian or Asian American, Pacific Islander, Hispanic or Latinx, Native American, multiracial, and other. More than half of these studies (*n* = 5) included samples with White youth as the majority, ranging from 40.90% White (Gray et al., 2024) to 90.00% White (Volungis, 2020). The other three studies included samples composed primarily of youth who identified as Hispanic (Kresovich et al., 2023 [74.00%]), or Black or African American (Ginsburg & Drake, 2002 [100%]; Melamed et al., 1978 [72.50%]). The remaining two studies that reported race and ethnicity were conducted in New Zealand (Fleming et al., 2016) and Singapore (Yeo et al., 2016), with the race and ethnicity options including Māori, Pacific Islander, New Zealand European, and other, and Chinese, Malay, Indian, and other, respectively. Fleming et al. (2016) had a majority Māori (34.10%) and Pacific Islander (27.30%) youth population, and Yeo et al. (2016) had a majority Chinese (68.70%) youth population. The remaining nine studies (47.37%) did not include any information about participants’ race or ethnicity and were conducted in the U.S. (*n* = 4), Australia (*n* = 2), Sweden (*n* = 1), and Indonesia (*n* = 1).

All studies reported additional demographic information distinct to their sample, such as participants’ religious breakdown, geographical location (e.g., suburban, urban), and school or district information. Notably, many of the 19 included studies reported participants’ clinical symptomology (*n* = 10; 52.63%), ranging from exhibiting symptoms of, previously diagnosed with, or meeting Diagnostic and Statistical Manual of Mental Disorders (DSM) criteria for diagnoses of anxiety disorder(s), depression, post-traumatic stress disorder, self-injurious behavior, and/or attention-deficit/hyperactivity disorder. Eight studies (42.10%) reported on participants’ socioeconomic status in some capacity (i.e., class category [‘low’, ‘middle’], percentage below the poverty line, percentage receiving free or reduced lunch, range and median of household income), while eleven studies did not. The relatively low proportion of studies reporting on socioeconomic status highlights another limitation in the current evidence base and further restricts our ability to understand the generalizability of findings across diverse populations.

### 3.3. Effectiveness

The 19 studies included in this scoping review evaluated evidence-based ingredients across outcomes, including anxiety (*n* = 13, 68.42%), depression (*n* = 7, 36.84%), suicidality (*n* = 3, 15.79%), and/or subjective wellbeing (*n* = 3, 15.79%). Notably, five studies assessed multiple target outcomes (i.e., depression and anxiety; subjective wellbeing, depression, and anxiety; depression and suicidality). We found that most studies (*n* = 13, 68.42%) used broader interventions that included more than one evidence-based ingredient (*M* = 3.63, *SD* = 2.69). In total, we identified 17 evidence-based ingredients. The following evidence-based ingredients were included across all of the reviewed manuscripts: mindfulness skills (*n* = 11), relaxation skills (*n* = 9), psychoeducation (*n* = 9), exposure (*n* = 6), cognitive restructuring (*n* = 6), social or interpersonal skills (*n* = 5), thought identification (*n* = 4), help-seeking skills (*n* = 3), emotion identification (*n* = 3), problem-solving skills (*n* = 3), activity scheduling (*n* = 2), modeling (*n* = 2), behavior analysis (*n* = 2), expressions of hope (*n* = 1), gratitude (*n* = 1), creating a trauma narrative (*n* = 1), and study skills (*n* = 1). In Appendix A, Table A1 summarizes each of the included studies’ findings, and Table A2 defines each of the evidence-based ingredients.

#### 3.3.1. Acceptability and Usability

Although the studies reported various results according to their specific research questions, the current scoping review focused on the results related to the effectiveness of the evidence-based ingredients in addressing anxiety, depression, suicidality, and/or subjective wellbeing. We found that three studies focused primarily on acceptability and usability of the evidence-based ingredients with overall positive results (i.e., anxiety: P. M. Barrett et al., 2001; Cannon et al., 2020; depression: Fleming et al., 2016). In one of these studies, youth and caregivers reported exposure with rewards as the most useful evidence-based ingredient for addressing anxiety, while relaxation skills and problem-solving skills were rated as less useful (P. M. Barrett et al., 2001). The other two acceptability studies found validation for practicing exposure with peer confederates (Cannon et al., 2020) and positive qualitative feedback for an internet-based intervention, though participants described it as more helpful for anger and challenging behavior than for depression (Fleming et al., 2016).

#### 3.3.2. Anxiety Outcomes

The remaining 16 studies examined the effects of evidence-based ingredients on anxiety, depression, suicidality, and/or subjective wellbeing. Of these, we identified 13 studies (81.25%) that reported significant improvements in at least one outcome from pre- to post-intervention. More specifically, seven of the 11 studies that measured anxiety found significant improvements in anxiety for participants who received the evidence-based ingredients compared to those in the control groups, while the other four studies did not. The most common evidence-based ingredients across those seven studies were relaxation skills (*n* = 4), exposure (*n* = 4), psychoeducation (*n* = 3), cognitive restructuring (*n* = 2), modeling (*n* = 2), and mindfulness skills (*n* = 2). Of the seven studies with significant reductions in anxiety, five reported on the effect size of this outcome. Four of those studies noted large effects (Bilek et al., 2022, Hedges’ *g* = 0.77; Ginsburg & Drake, 2002, partial eta-squared = 0.28–0.54; Rudy et al., 2017, Cohen’s *d* = 2.39–3.18; Schibbye et al., 2024, Cohen’s *d* = 1.00–1.60), whereas Yeo et al. (2016) noted a medium effect size (partial eta squared = 0.12). Two of the four studies that did not yield statistically significant effects reported effect sizes to evaluate clinical relevance, both of which were small (Tol et al., 2008, Cohen’s *d* = 0.14; Gray et al., 2024, Cohen’s *d* = 0.19). Additionally, we noticed that two of the four studies that did not find significant improvements in anxiety directly after the intervention found significant improvements in anxiety at the follow-up (Gray et al., 2024; see maintenance section) or found generally positive results without reporting the statistical significance of those results (Jennings & Jennings, 2013).

#### 3.3.3. Depression Outcomes

For the six studies that measured depression, all but one of them found significant improvements in depression for participants who received the evidence-based ingredients compared to their baseline levels (*n* = 2) or to those in the control groups (*n* = 3). However, we recognized caveats for three of the five studies. One study found improvements in participants’ knowledge around depression rather than depressive symptoms (Volungis, 2020). Another study only found reductions in depression for participants with lower academic achievement (Kresovich et al., 2023). The third study found improvements in depression through an indirect pathway involving increased nonjudgment and decreased rumination (Hilt et al., 2024). The most common evidence-based ingredients across all five studies were mindfulness skills (*n* = 4), psychoeducation (*n* = 3), social or interpersonal skills (*n* = 2), and relaxation skills (*n* = 2). Of the five studies with significant improvements in depression, only two reported on the effect size of this outcome, which were small (Joyce et al., 2010, Cohen’s *d* = 0.27; Tol et al., 2008, Cohen’s *d* = 0.31). Although Joyce et al. (2010) found medium to large effects for those with higher baseline scores (i.e., borderline, abnormal; Cohen’s *d* = 0.62). The study that did not find significant improvements reported effect sizes to evaluate clinical relevance, which were very small (Gray et al., 2024, Cohen’s *d* = 0.09).

#### 3.3.4. Suicidality Outcomes

We found three studies that measured suicidality, of which two found significant improvements in participants’ knowledge, awareness, and attitudes around suicide for those who received the evidence-based ingredients, and one found no significant differences. The most common evidence-based ingredients across the three studies that measured suicidality were psychoeducation (*n* = 3), social or interpersonal skills (*n* = 3), and help-seeking skills (*n* = 2). Of the three studies that measured suicidality, none of them reported on the effect sizes of their outcomes (Kalafat & Elias, 1994; Kresovich et al., 2023; Volungis, 2020). In addition to these studies, Gray et al. (2024) reported an increase in the number of participants who endorsed suicidal ideation following evidence-based ingredient delivery compared to baseline; however, this was based on a single item within a broader depression measure and was only reported descriptively (i.e., total count, percentage of sample) with no significance indicated.

#### 3.3.5. Subjective Wellbeing Outcomes

All three studies that measured subjective wellbeing found significant improvements in the subjective wellbeing domains of gratitude, hope, resilience, and self-compassion for participants who received the evidence-based ingredients compared to their baseline levels (*n* = 1) or to those in the control groups (*n* = 2). We noticed that in one study, improvements in resilience and self-compassion were only evident for participants with lower academic achievement (Kresovich et al., 2023). There were no significant findings related to life satisfaction from baseline to post-implementation, but there were from post-implementation to follow-up (Swierad & Williams, 2023; see Section 3.6). The most common evidence-based ingredients across the three studies were mindfulness skills (*n* = 3), relaxation skills (*n* = 2), psychoeducation (*n* = 2), and emotion identification (*n* = 2). Of these three studies targeting subjective wellbeing, only one reported on the effect size of their outcome, which was a small effect for hope (Tol et al., 2008, Cohen’s *d* = 0.29).

#### 3.3.6. Moderators and Reporting Effects

Across studies, only three (18.75%) explicitly examined whether effects varied across participant characteristics (Kresovich et al., 2023; see maintenance section for Yeo et al., 2016 and Hilt et al., 2024). These analyses explored baseline symptomology or academic achievement as moderators. Overall, subgroup findings were limited and inconsistent, with no studies systematically testing differences in effectiveness by race, ethnicity, or gender. Thus, it remains unclear whether evidence-based ingredients function similarly across diverse youth populations. Additionally, only half of the sixteen studies examining effectiveness reported the magnitude of their results. There was inconsistent reporting of effect sizes across outcomes, with many studies limiting effect size information to significant findings. Among studies measuring anxiety that reported effect sizes, most reported large effects, though two non-significant studies reported small effects. In contrast, studies measuring depression that reported effect sizes generally found small effect sizes, with one noting stronger effects (i.e., medium to large) among youth with higher baseline symptoms. No studies measuring suicidality reported effect sizes, and only one study measuring subjective wellbeing reported effect sizes, with small effects. Overall, the reported effects ranged from small to large, highlighting variability in both reporting practices and ingredient impact across studies.

### 3.4. Adoption

The paraprofessionals who delivered the evidence-based ingredients included graduate students (*n* = 4; 21.05%; two studies specified graduate students in psychology), teachers (*n* = 4; 21.05%), the youth themselves (*n* = 3; 15.79%), peers (*n* = 3; 15.79%), caregivers (*n* = 2; 10.53%), nurses (*n* = 1; 5.26%), and unspecified local paraprofessionals (*n* = 1; 5.26%). Two of the studies (10.53%) used recordings to deliver the evidence-based ingredients (Heitkemper et al., 1993; Melamed et al., 1978). Paraprofessionals delivered the evidence-based ingredients in schools (*n* = 11, 57.89%), on the internet (*n* = 5; 26.32%), at a dentist’s office (*n* = 3; 15.79%), or in a university laboratory or clinic (*n* = 2; 10.53%). See Appendix A Table A1 for a detailed breakdown of delivery with citations.

Training of paraprofessionals varied widely across studies and was often inconsistently reported. Some studies described structured training procedures, including didactic instruction on the intervention, modeling of evidence-based ingredients, and opportunities for practice (e.g., role-play), sometimes accompanied by supervision or ongoing support. Other studies provided limited details regarding training procedures or did not report training at all. In some cases, differences in training approaches appeared to correspond to variations in paraprofessional background (e.g., graduate students versus teachers, peers, or caregivers); however, these patterns were not systematically examined. Due to this variability and limited reporting, it remains unclear which training components are most critical for the effective delivery of evidence-based ingredients.

Lastly, two studies (10.53%) did not specify the setting of delivery (P. M. Barrett et al., 2001; Bilek et al., 2022). Four studies (21.05%) involved multiple settings, which included a combination of the internet with either the classroom (Fleming et al., 2016; Kresovich et al., 2023; Swierad & Williams, 2023) or their home and dentist office (Schibbye et al., 2024).

### 3.5. Implementation

Across settings, the 19 included studies varied in how their paraprofessionals delivered the evidence-based ingredients and the associated costs (e.g., time, resources). Most commonly, paraprofessionals delivered evidence-based ingredients in a weekly session format (*n* = 9; 47.37%). Other studies used single sessions (*n* = 3; 15.79%), biweekly or triweekly sessions (*n* = 2; 10.53%), daily exercises (*n* = 1; 5.26%), or two-day programs (*n* = 1; 5.26%). Two studies (10.53%) provided broad descriptions (i.e., progress through seven game modules, flexible classroom lessons) without specifying the delivery format (Fleming et al., 2016; Joyce et al., 2010). One study (5.26%) did not report on format delivery (Cannon et al., 2020). The average number of sessions (i.e., single instances in which an evidence-based ingredient was delivered, whether that be one-hour long or one-day long) was approximately 10.31 (*SD* = 14.80), with a range of a single session to 63 total sessions. Notably, the 63 sessions were brief, 10 min daily exercises delivered three times per day (Hilt et al., 2024). On average, the total duration of delivering the evidence-based ingredients was 343 min (*SD* = 280.85 min) and ranged from 10 min to 840 min total. See Appendix A Table A1 for a detailed breakdown of implementation with citations.

Despite differences in delivery format and duration, most of the studies (*n* = 14; 73.68%) provided training for their paraprofessionals on the evidence-based ingredients, which included initial training (*n* = 12), ongoing training (*n* = 5), and fidelity checks for implementation (*n* = 3). Most studies (*n* = 12; 63.16%) also mentioned providing supervisors for their paraprofessionals to support the delivery of evidence-based ingredients. Supervisors varied greatly across studies, with licensed psychologists, social workers, curriculum or manual authors or instructors, research assistants, post-doctoral therapists, experienced meditators, and school counselors serving as supervisors. One study mentioned supervision but did not specify who served as the supervisor (Ginsburg & Drake, 2002). Supervisors’ roles included delivering the initial training (*n* = 6, 50.00%), providing ongoing training and support (*n* = 5, 41.67%), administering fidelity checks (*n* = 2; 16.67%), and/or overseeing youth’s self-administration (e.g., providing tech support, ensuring they are on-task completing the modules on the internet; *n* = 2; 16.67%). All 19 studies explained that certain resources (i.e., worksheets, recordings, curriculum or intervention manual, application, website) were required for implementing their evidence-based ingredients.

Across studies, the adequacy of training and supervision could not be determined due to limited reporting detail. Only three studies (15.79%) included fidelity checks, limiting confidence in the consistency or quality of implementation. The variability across studies suggests that the feasibility and intensity of training, supervision, and support differ by paraprofessional type. For example, graduate students often had prior coursework or clinical training and therefore required less intensive oversight, relying instead on structured supervision or fidelity check-ins to maintain adherence. Teachers typically participated in shorter workshops or professional development sessions that emphasized integration of ingredients into classroom routines, with variable ongoing support. Studies involving peers or self-administering youth generally used highly structured manuals, scripted activities, or digital scaffolding paired with brief researcher check-ins to promote consistency. These differences indicate that feasibility, scalability, and training needs for paraprofessional-delivered evidence-based ingredients are likely contingent on paraprofessionals’ baseline skill levels and access to supervision resources, underscoring the importance of tailoring preparation and support to each role.

### 3.6. Maintenance

Although most studies (*n* = 13, 68.42%) did not report on the long-term impact of evidence-based ingredients on youth outcomes, six studies (31.58%) included follow-up results that ranged from one week to six months after the evidence-based ingredients were delivered. These studies examined youth outcomes related to anxiety (*n* = 4), depression (*n* = 3), and/or subjective wellbeing (*n* = 2). While none of these studies evaluated suicidality as their primary outcome, one study (Gray et al., 2024) that targeted anxiety and depression also reported changes over time in a suicidal ideation item embedded within their depression measure. See Appendix A Table A1 for a detailed breakdown of maintenance with citations.

Among the four studies measuring anxiety, half found significant reductions in anxiety at the two-month follow-ups with large effect sizes (Gray et al., 2024, [Cohen’s *d* = 0.92]; Yeo et al., 2016), particularly among participants with higher baseline anxiety (Yeo et al., 2016, [partial eta-squared = 0.28–0.29]). The other two studies found no significant changes (Rudy et al., 2017 [one month; no effect size reported]; Tol et al., 2008 [six months; Cohen’s *d* = 0.04]), though one reported a downward trend in anxiety over time (Rudy et al., 2017). For the three studies measuring depression, one study (Hilt et al., 2024) found significant decreases in depression through a serial mediation pathway, where increased nonjudgment and reduced rumination over time decreased depressive symptoms at the 12-week and six-month follow-ups. The two other studies found no significant differences in depression at the follow-ups with observations of a sustained or slightly diminishing effect (Gray et al., 2024 [two months; Cohen’s *d* = 0.54]; Tol et al., 2008 [six months; Cohen’s *d* = 0.24]). Notably, within their measure of depression, Gray et al. (2024) observed a decrease in the number of participants who endorsed recent suicidal ideation at the follow-up that fell below baseline endorsement, despite an initial increase directly following the intervention. Notably, these were simply reported as a count of the sample and observed trends, not significant differences, from a single item on the broader depression measure.

Two studies measured subjective wellbeing and specifically examined life satisfaction, gratitude (Swierad & Williams, 2023), and hope (Tol et al., 2008). These studies found significant improvements in life satisfaction (from post-implementation to one-week follow-up; no effect size reported) and gratitude (from baseline to one-week follow-up, gains maintained post-implementation; no effect size reported); however, no significant changes were found in hope (six-month follow-up). Despite the lack of statistical significance, individuals’ levels of hope immediately after delivery were maintained at the follow-up for those who received the evidence-based ingredients, whereas hope declined for those in the control group, resulting in a moderate effect size that increased over time (Cohen’s *d* = 0.38; Tol et al., 2008). Across all studies, follow-up assessments were limited and varied in timing (one week to six months) and reporting of effect size, which constrains conclusions about the overall maintenance of effects.

## 4. Discussion

This scoping review investigated the current literature on social-emotional evidence-based ingredients delivered by paraprofessionals to support youth mental health and wellbeing. Most studies measured youth outcomes related to anxiety and depression, with a smaller number of studies measuring suicidality and subjective wellbeing. The majority of the studies used interventions that included multiple evidence-based ingredients. Across all studies, 17 evidence-based ingredients were identified: mindfulness skills, relaxation skills, psychoeducation, exposure, cognitive restructuring, social or interpersonal skills, thought identification, help-seeking skills, emotion identification, problem-solving skills, activity scheduling, modeling, behavior analysis, expressions of hope, gratitude, creating a trauma narrative, and study skills. Paraprofessionals included graduate students, teachers, peers, nurses, caregivers, unspecified local paraprofessionals, and the youth themselves (i.e., self-administration). Most commonly, paraprofessionals delivered these evidence-based ingredients in schools or community settings through weekly sessions over multiple weeks. However, delivery methods varied among studies, ranging from a single session to three daily exercises over several weeks. Further, some studies used self-administration where youth received evidence-based ingredients through the internet (e.g., recordings, applications, website modules). In terms of training and support, most studies described training their paraprofessionals in some capacity (e.g., initial, ongoing) and providing necessary resources for implementation (e.g., manuals, worksheets, videos). Many studies also designated supervisors to support paraprofessionals in their delivery; however, the degree of support (e.g., fidelity checks, ongoing training, weekly debrief meetings) varied across studies. This variability highlights a central implementation challenge for school behavioral health systems: identifying what types and levels of implementation support are sufficient to ensure safe, effective, and sustainable delivery of evidence-based ingredients by paraprofessionals.

In terms of effectiveness, more than two-thirds of the 19 included studies demonstrated significant improvements in at least one of their targets of interest from before and after the delivery of evidence-based ingredients. These studies found significant reductions in anxiety and depressive symptoms, and significant increases in subjective wellbeing and knowledge and attitudes around suicide. Additionally, a few studies focused solely on the acceptability and usability of evidence-based ingredients rather than their effectiveness and found favorable youth and caregiver feedback. The significant improvements and favorable feedback observed in most studies suggest that paraprofessionals can feasibly and successfully deliver evidence-based ingredients to support youth mental health and wellbeing. However, studies inconsistently reported effect sizes, and those that did revealed wide variation in magnitude across outcomes (i.e., small to large). This suggests that while paraprofessionals can effectively deliver evidence-based ingredients, the strength and consistency of these effects remain unclear. From an implementation perspective, this inconsistency underscores the need for clearer reporting standards and stronger link between implementation supports (e.g., training dosage, coaching models) and outcome strength. Future researchers should systematically report and compare effect sizes to better understand the practical impact of paraprofessional-delivered ingredients.

Across studies, several evidence-based ingredients consistently emerged as contributing to these positive outcomes, particularly mindfulness skills, relaxation skills, psychoeducation, exposure, cognitive restructuring, social or interpersonal skills, and thought identification. Notably, although several of these ingredients were associated with improvements in depressive outcomes, behavioral activation (i.e., activity scheduling) was relatively underrepresented in the included studies despite being a well-established intervention for depression (Malik et al., 2021; Ritschel et al., 2016). This discrepancy highlights a potential gap between the broader evidence base and the ingredients currently being implemented by paraprofessionals and underscores the need for greater emphasis on training in behavioral activation strategies. Despite this gap, the ingredients that were most commonly implemented still align with broader developmental and clinical frameworks, reflecting core processes that promote adaptive emotion regulation, cognitive flexibility, and social functioning. From a bioecological framework (Bronfenbrenner & Morris, 2006), paraprofessionals become a part of youths’ proximal system, fostering meaningful interactions through which development occurs. Embedding evidence-based ingredients within these everyday contexts aligns with MTSS frameworks, which emphasize prevention and early intervention delivered by school-based personnel. As none of the included studies explicitly reported tier classifications, we inferred tier based on intervention scope, target population, and delivery context. Interventions delivered in a general educational setting (e.g., embedded in classroom instruction, school-wide prevention programming, SEL curricula) could be considered as Tier 1, or universal, support (i.e., Gray et al., 2024; Jennings & Jennings, 2013; Joyce et al., 2010; Kalafat & Elias, 1994; Kresovich et al., 2023; Swierad & Williams, 2023; Volungis, 2020; Yeo et al., 2016). In contrast, interventions targeting youth with elevated symptoms or diagnoses could be considered as Tier 2 and Tier 3 support, or targeted and increasingly intensive small group or individualized support, (i.e., Ginsburg & Drake, 2002; Tol et al., 2008); however, contextual factors should also be considered when determining the appropriate tier for delivery (e.g., Tier 1 delivery of mental health intervention for high-risk or trauma-exposed communities where *all* students would benefit; Tol et al., 2008). Interestingly, one study (i.e., Fleming et al., 2016) designed their intervention as targeted support (i.e., Tier 2 or 3), but participant feedback suggested potential acceptability as universal programming (i.e., Tier 1), highlighting the fluidity of tier classification depending on implementation context. Further, consistent with the Positive Youth Development perspective (Benson et al., 2004; Benson et al., 2011; Lerner et al., 2011), these interactions with skill-building opportunities cultivate developmental assets (e.g., competence, confidence, connection) that foster resilience and positive development. More specifically, the identified ingredients reflect transdiagnostic, third-wave therapeutic approaches (e.g., cognitive-behavioral therapy, acceptance and commitment therapy) that target underlying mechanisms of youth mental health difficulties, such as one’s relationship with their maladaptive cognitions, emotion dysregulation, and social skill deficits, to promote adaptive coping and resilience. Taken together, these frameworks clarify how paraprofessionals can promote youth wellbeing by delivering targeted skills.

These findings suggest that a range of non-licensed school-based and community-based personnel may be able to deliver specific evidence-based ingredients when appropriate implementation supports are in place. However, the paraprofessional roles identified in this review varied substantially in their background, training, and capacity to deliver these ingredients, which has important implications for implementation. For example, psychology graduate students may require less extensive initial training due to prior knowledge of psychological concepts, whereas teachers may need additional instruction to orient them to non-academic intervention skills and adapt them to the classroom context. In contrast, peers, caregivers, and youth may require more structured guidance, training, and ongoing supervision to ensure fidelity, given that they often do not have formal training in psychology, intervention delivery, or helping skills.

These differences in training needs also align with the complexity of the ingredients being delivered. Some ingredients (e.g., mindfulness skills, relaxation skills, psychoeducation) may be more readily implemented by paraprofessionals with relatively brief training, whereas others (e.g., cognitive restructuring, exposure) likely require more intensive training, coaching, and supervision to ensure fidelity and minimize risk. This distinction has direct implications for how school behavioral health systems allocate personnel and training resources and design tiered implementation support models.

While many studies reported positive short-term effects, few examined the mechanisms or sustainability of these outcomes. Only three studies explored moderators (i.e., academic achievement, baseline severity) or mediators (i.e., changes in nonjudgment and rumination). Additionally, less than one-third of the studies reported the long-term outcomes of evidence-based ingredients, and their findings were mixed. Some studies demonstrated novel or maintained benefits at follow-up, while others showed no significant change over time or only trends towards improvement. Taken together, these findings provide preliminary evidence that paraprofessional-delivered evidence-based ingredients may contribute to short-term improvements in youth outcomes; however, the limited number of studies, variability in effect sizes, and inconsistent reporting warrant caution in interpreting these findings. Long-term outcomes remain variable and unclear. Future research is needed to clarify for whom and under what circumstances these benefits are sustained over time. For school behavioral health systems, more information is needed on how training intensity and supervision relate to maintenance of outcomes across MTSS tiers.

### 4.1. Implications

The findings from this scoping review provide support that paraprofessionals can deliver informal mental health care grounded in evidence-based practices and that many young people demonstrate positive short-term outcomes as a result. The common pattern of training and supervision across studies suggests that paraprofessionals seem to most effectively provide this support through task-shifting models that incorporate supervision and training from specialists to aid in their delivery. As paraprofessionals help foster coping skills in youth, highly trained clinicians can focus their limited time on youth with higher support needs that may not be addressed through coping skills. This task-shifting approach leverages paraprofessionals to expand mental health services to efficiently and effectively meet the rising mental health needs of youth. These findings are particularly relevant given the ongoing youth mental health crisis (McGorry et al., 2025), workforce shortages (Butryn et al., 2017; National Center for Health Workforce Analysis, 2024), and barriers to formal mental health care (see Anderson et al., 2017). Informal, community-based care from paraprofessionals through task-shifting can increase the number of personnel who effectively provide support to youth while simultaneously diminishing barriers associated with formal care (e.g., high costs, insurance, long waitlists).

Rather than privileging a single workforce role, the current findings suggest that schools may benefit from adopting flexible workforce models in which a range of paraprofessionals, such as teachers, teaching assistants, one-on-one aides, mentors, peers, caregivers, and community partners, deliver selected evidence-based ingredients under supervision. This aligns with interconnected systems frameworks that emphasize collaboration between co-occurring support systems in schools (e.g., positive behavioral supports and interventions, community mental health providers, academic supports) to create a more comprehensive, coordinated system of care (Splett et al., 2017; Weist et al., 2001; Zabek et al., 2023). However, in order for schools to successfully integrate paraprofessionals into mental health service delivery, intentional implementation supports (e.g., practice-based coaching, systems-level consultation) are critical for the adoption of task-shifting, workforce capacity, fidelity of services provided, and sustainability within dynamic school behavioral health systems.

One promising, underutilized paraprofessional for task-shifting is mentors (S. D. McQuillin et al., 2022). Mentors are well-positioned community members who are already embedded in the daily lives and microsystems of youth to support positive youth development (DuBois et al., 2011; Raposa et al., 2019). Further, mentors have pre-existing safe, trusting relationships with youth that serve as strong foundations for integrating and fostering coping skills (Keller, 2005; Morrow & Styles, 1995). Prior literature (e.g., Cavell et al., 2021; Lyons et al., 2019; Werntz et al., 2023) indicates that quality mentoring relationships balanced with targeted activities tend to yield greater youth outcomes. The findings from this review suggest that evidence-based ingredients may serve as an effective targeted activity to enhance youth outcomes related to mental health and wellbeing. Although the term ‘mentor’ was not explicitly used in the included studies, some paraprofessionals within the studies are commonly considered informal mentors (e.g., teachers). Further, many studies detailed their training and promoted the notion that other paraprofessionals, such as mentors, could be trained to implement their interventions. To maintain the effectiveness of evidence-based ingredients provided by mentors, training and supervision from specialists through a task-shifting model is necessary. Mentors should receive structured training and supervision on how to deliver specific evidence-based ingredients. Whereas the current review encountered wide variations in the level of training and supervision provided, prior literature suggests that ongoing training and supervision are critical for effective implementation and outcomes, particularly when mentors are supporting youth from diverse cultural backgrounds or discussing experiences related to discrimination and inequity (Flitner, 2023; S. D. McQuillin & Lyons, 2021; Simpson et al., 2023). Future research should focus on optimizing mentor training for evidence-based ingredients and developing brief, modular interventions rooted in distinct evidence-based ingredients that can be flexibly applied within mentoring relationships. Across paraprofessional roles, the findings underscore the importance of tailoring implementation support to the complexity of specific ingredients. Future research should focus on identifying minimum effective training and supervision requirements for ingredients and testing scalable coaching models that can be embedded within school behavioral health systems. In addition, expanding the behavioral health workforce through training undergraduate students in evidence-based ingredients represents a promising task-shifting strategy, as emerging training models demonstrate that undergraduate students can be equipped with applied, supervised skills to support youth in school and community settings (e.g., Ballmer Institute for Children’s Behavioral Health, University of Oregon).

### 4.2. Limitations

The results of this scoping review reveal promise for task-shifting evidence-based ingredients to mentors and other paraprofessionals to expand school behavioral health care. However, limitations in the screening and data extraction processes were present. First, the focused inclusion criteria, which prioritized studies examining discrete evidence-based ingredients delivered by paraprofessionals, may have excluded relevant studies and limited the breadth of the evidence base. Second, only one person (i.e., the first author) screened and excluded articles based on the eligibility criteria and coded for evidence-based ingredients. Without additional coders included in the process, there is potential for selection bias and subjective decision-making that influence the inclusion and exclusion of articles and the identification of evidence-based ingredients. This may have limited the reliability of the synthesis, particularly in capturing subtle differences in how evidence-based ingredients were defined, operationalized, or grouped across studies. Further, some studies reported ambiguous or brief descriptions of their treatment that made it difficult to ensure that all evidence-based ingredients within their broader intervention were captured. Future reviews should incorporate additional independent reviewers to strengthen the reliability of the selection and identification process. Additionally, the search strategy was limited to two databases (PsycINFO and PubMed) and did not include grey literature or citation tracking procedures. As a result, some relevant studies—particularly unpublished evaluations or program reports describing feasible paraprofessional-delivered ingredients—may not have been captured, which may further limit the comprehensiveness of the review. The current review was also limited to articles published in English, which may have excluded relevant studies published in other languages and thus limits the generalizability of the conclusions.

Further, this review assumed a degree of invariance across study samples when interpreting results, despite the notable differences in participants’ age, gender/sex, race and ethnicity, nationality, and clinical diagnoses. Only three studies examined subgroup differences in effectiveness, and these were limited to comparisons by baseline symptom severity or academic achievement rather than by race, ethnicity, socioeconomic status, or gender identity. Studies’ inconsistent reporting on participant demographics, particularly race, ethnicity, and socioeconomic status, further limits our ability to assess whether evidence-based ingredients are effective across diverse populations and restricts equitable application in school and community settings. Future research should adopt more systematic demographic reporting and test whether intervention effects vary across demographic and clinical subgroups to clarify for whom and under what conditions these evidence-based ingredients are most effective. Finally, most of the included studies evaluated interventions that encompassed multiple evidence-based ingredients and did not isolate or analyze the effects of individual evidence-based ingredients, limiting our ability to determine which are most effective for improving targeted outcomes. Future research should prioritize designs that examine discrete evidence-based ingredients or conduct analyses that identify the unique impact of each ingredient.

### 4.3. Strengths and Conclusions

Nevertheless, this scoping review also demonstrated several strengths that reinforce its relevance for school mental health and mentoring. This was the first scoping review, to our knowledge, that investigated the delivery of evidence-based ingredients by paraprofessionals specifically for youth. Although prior research (e.g., Patel et al., 2017; Singla et al., 2021) has demonstrated support for paraprofessionals delivering evidence-based practices to adults, less research has focused on whether the same holds true for youth. This review advances our understanding of how paraprofessionals can effectively provide evidence-based care to youth, indicating that the use of paraprofessionals may offer a promising approach for school-based prevention and early intervention of mental health challenges. Further, this review shifted the focus away from delivering entire treatment packages (i.e., evidence-based practices) and towards transdiagnostic, evidence-based *ingredients* (i.e., specific skills, active components). This shift may allow for more flexible, scalable approaches, which are especially important for low-resource settings and informal care. The focus on discrete skills allows for integration into nonclinical settings, such as schools and mentoring programs, that may have less dedicated time for intensive mental health care. Another strength of this review is its international scope. This review examined studies across six different countries (i.e., Australia, Indonesia, New Zealand, Singapore, Sweden, U.S.) suggesting the global relevance of expanding youth mental health care by task-shifting to paraprofessionals across diverse sociocultural contexts.

Overall, this review demonstrated that task-shifting evidence-based ingredients to paraprofessionals is a feasible, promising, and scalable approach for extending and strengthening youth mental health supports. The findings from this review highlight the importance of building capacity in the paraprofessional workforce (e.g., optimizing training and supervision) and leveraging paraprofessionals in school behavioral health support systems. Future research should focus on identifying effective implementation support models, particularly training and coaching strategies, that enable schools to safely and equitably scale evidence-based psychosocial practices across tiers of support.

## Figures and Tables

**Table 1:** Detailed Eligibility Criteria.

	Inclusionary Criteria	Exclusionary Criteria
Study Design	The study is an original research study published in a peer-reviewed journal.	The study is a meta-analysis, review paper, commentary, case study, single-case design, pre-published study, or dissertation.
Language	The study is published or translated into English.	The study is published in a language other than English.
Participant Demographics	All participants in the study are youth below 18 years of age.	Some or all participants in the study are adults above 18 years of age.
Variables of Interest	The study includes a youth-focused intervention that has a distinct evidence-based ingredient(s). The intervention or ingredient is delivered by a paraprofessional (i.e., individuals without a formal license). The study evaluates the impact of the intervention/ingredient(s) on anxiety, depression, suicidality, or subjective wellbeing.	The study does *not* include a youth-focused intervention and/or the youth-focused intervention is multi-faceted and does *not* have a distinct evidence-based ingredient(s). The intervention or ingredient is *not* delivered by a paraprofessional (i.e., delivered by a professional with a formal mental health license). The study does *not* evaluate the impact of the intervention/ingredient(s) on anxiety, depression, suicidality, or subjective wellbeing.

**Table 2:** Search Strategies and Terms.

Search	Search Terms	PsycINFO Results	PubMed Results
S1	youth* OR child* OR adolescen* OR “early adolescen*” OR teen* OR kid* OR juvenile* OR “elementary school*” OR “middle school*” OR “high school*” OR student*	1,969,250	5,156,342
S2	anxiety OR anxious OR depression OR depressive OR chedul* OR “subjective wellbeing” OR “subjective wellbeing” OR “life satisfaction” OR “quality of life” OR “positive affect”	801,586	1,382,606
S3	relax* OR calm* OR ground* OR breath* OR chedule* OR mindful* OR “progressive muscle relax*” OR “body scan*” OR “cognitive chedulere*” OR “self-monitor*” OR “emotion* awareness” OR “emotion* regulat*” OR “thought awareness” OR “though trap*” OR exposure OR habituat* OR “facing fear*” OR “face your fear*” OR “behavioral activation” OR “activity chedule*” OR “behavioural activation” OR “social-emotional” OR “social emotional” OR “self-awareness” OR “self-management” OR “social awareness” OR “relationship skill*” OR “responsible decision-mak*”	444,054	2,068,098
S4	mentor* OR lay* OR paraprofessional* OR “community health worker*” OR peer* OR “non-mental health professional*” OR “non-specialist health work*” OR “support specialist*” OR coach*	261,061	328,653
S5	brief OR “brief treatment” OR “single session intervention” OR kernel OR element OR component OR ingredient OR module	536,243	4,188,545
S1 AND S2		280,703	339,479
S1 AND S2 AND S3		36,387	37,299
S1 AND S2 AND S3 AND S4		2863	2247
S1 AND S2 AND S3 AND S4 AND S5		426	270
S1 AND S2 AND S3 AND S4 AND S5 (PsycINFO and PubMed Limiters: English)		419	266
S1 AND S2 AND S3 AND S4 AND S5 (PsycINFO Limiters: English, Peer Reviewed; Pub Med Limiters: English, Clinical Trial)		318	38
S1 AND S2 AND S3 AND S4 AND S5 (PsycINFO Limiters: English, Peer Reviewed, Age < 18; Pub Med Limiters: English, Clinical Trial, Age < 18)		176	33

## Data Availability

Not applicable. The original contributions presented in this study are included in the article. Further inquiries can be directed to the corresponding author(s).

## Appendix A

**Table A1:** Chart of Descriptives, Demographics, Evidence-Based Ingredients, & Outcomes.

Study	Sample	Measured	Ingredient(s)	Youth Outcomes	Delivery	Training
**Author(s) (Year):** P. M. Barrett et al. (2001) **Study Design:** Quasi-experimental **Broader intervention:** FRIENDS program	**Size:** *N* = 48 **Age Range:** N/A **Age M (SD):** 10.26 (1.12) **Gender:** 61.67% female, 38.33% male **Race/Ethnicity:** N/A **Location:** Australia	Anxiety	Mindfulness skills, thought identification, cognitive restructuring, relaxation skills, problem-solving skills, exposure	Caregivers of children and adolescents rated the FRIENDS program positively. Caregivers rated introductions and normalization of anxiety, and identifying/communicating emotions, relaxation games, and problem-solving plans as the least useful skills (*p* < 0.01); and self-rewarding for graduated exposure as the most useful (*p* < 0.01). Around 98% of youth reported they found the skills fun/okay and would use them again often/sometimes. Youth rated graduated exposure and self-rewarding for graduated exposures as the most useful skills (*p* < 0.01). No significant correlations were found between treatment evaluations and clinical outcomes.	**Who:** graduate students **Setting:** N/A **Supervisor:** N/A ***Supervisor role:*** *N/A* **Duration:** 10 weekly sessions, 2 booster sessions; 70 min each	Fidelity checks from videotaped sessions.
**Author(s) (Year):** Bilek et al. (2022) **Study Design:** Randomized Controlled Trial (RCT) **Broader intervention:** Exposure-focused CBT [vs. relaxation mentorship training control]	**Size:** *N* = 102 **Age Range:** 7–17 **Age M (SD):** 11.90 (3.10) **Gender:** 74.50% female, 25.50% male **Race/Ethnicity:** 76.47% White, 14.71% Multiracial, 3.92% Black, 3.92% Asian, 0.98% other or do not wish to identify **Location:** Michigan	Anxiety	Exposure	Treatment response was more likely in the exposure condition than the relaxation control condition (*Β* = 1.73 ± 0.66, *p* = 0.01). Anxiety scores decreased significantly for both conditions, but improvement was faster and more pronounced for exposure (decrease of 0.58 points/week; *SE* = 0.05, *p* < 0.001) than control (decrease of 0.26 points/week; *SE* = 0.07, *p* < 0.001). The exposure condition outperformed the relaxation control condition for anxiety scores at week 6 (*M_diff_* = 1.80 points, *p* < 0.05), 9 (*M_diff_* = 2.70 points, *p* < 0.01), and 12 (*M_diff_* = 3.60 points, *p* < 0.001, g = 0.77).	**Who:** graduate students **Setting:** N/A **Supervisor:** licensed psychologist or social worker with established CBT experience ***Supervisor role:*** fidelity checks **Duration:** 12 weekly sessions; 45–60 min each	Initial training. Fidelity checks from audiotaped sessions.
**Author(s) (Year):** Cannon et al. (2020) **Study Design:** Observational, proof-of-concept **Broader intervention:** Unfamiliar Peers Paradigm	**Size:** *N* = 105 **Age Range:** 14–15 **Age M (SD):** N/A **Gender:** N/A **Race/Ethnicity:** N/A **Location:** Maryland, Virginia, Washington, D.C.	Anxiety (social)	Exposure	Simulated exposures showed strong acceptability and usability for assessing adolescent social anxiety, social skills, and distress in ecologically valid ways. Peer confederate reports aligned with observer ratings and adolescent self-reports (*r* = 0.44–0.59, *p* < 0.001) and predicted distress beyond baseline arousal. Self-reported safety behaviors and standardized measures correlated with observer ratings and distress (*r* = 0.39–0.67), with adolescents above the clinical cutoff showing significantly worse functioning (*d* = 0.69–1.16). Minimal habituation was observed, with primacy effects indicating reduced distress only for the first task (*p* < 0.05).	**Who:** research personnel & peer confederate **Setting:** university laboratory **Supervisor:** N/A ***Supervisor role:*** N/A **Duration:** 3 tasks; 20 min total	Initial training (~15 h).
**Author(s) (Year):** Fleming et al. (2016) **Study Design:** Qualitative **Broader intervention:** computerized CBT (cCBT) SPARX program	**Size:** *N* = 39 **Age Range:** 13–16 **Age M (SD):** 14.50 (0.76) **Gender:** 38.46% female, 61.54% male **Race/Ethnicity:** 34.10% Māori, 27.30% Pacific Islander, 22.70% New Zealand European, 4.55% other, 11.40% no response **Location:** New Zealand	Depression	Thought identification, cognitive restructuring, relaxation skills, problem-solving skills, psychoeducation, expressions of hope, activity scheduling, social skills, mindfulness skills	Around 85% of participants expressed cCBT as helpful and enjoyable. Participants rarely used terms like “depression” in describing their experiences and instead emphasized changes in anger and behavior (e.g., feeling calmer, less angry, fighting less). Common qualitative themes included: (1) preferring cCBT over traditional counseling; (2) believing that using cCBT could increase help-seeking behavior; (3) support for offering cCBT universally.	**Who:** youth self-administered online **Setting:** classroom, internet **Supervisor:** alternative education tutor ***Supervisor role:*** oversee self-administration **Duration:** 7 game levels; 30 min each	N/A
**Author(s) (Year):** Ginsburg and Drake (2002) **Study Design:** RCT pilot **Broader intervention:** School-based CBT	**Size:** *N* = 12 **Age Range:** 14–17 **Age M (SD):** 15.60 (N/A) **Gender:** 83.33% female, 16.67% male **Race/Ethnicity:** 100% African American **Location:** Baltimore	Anxiety	Cognitive restructuring, relaxation skills, exposure, psychoeducation, social skills	75% of participants in CBT no longer met criteria for their primary anxiety disorder after treatment, compared to 20% in the control. Although both groups had lower anxiety impairment/severity scores (i.e., ADIS-CIR) post-treatment (*F*_1,7_ = 10.47, *p* < 0.01), participants in CBT showed greater reductions (*M_pre_* = 7.25, *M_post_* = 1.75, *d* = 2.70) than the control (*M_pre_* = 6.20, *M_post_* = 5.00, *d* = 0.56), *F*_1,7_ = 3.88, *p* < 0.05. Participants in both CBT and control had lower social anxiety scores (i.e., SAS-A) post-treatment (CBT: *M_pre_* = 54.50, *M_post_* = 44.00, *d* = 0.54; control: *M_pre_* = 51.75, *M_post_* = 46.25, *d* = 0.28), *F*_1,7_ = 8.49, *p* < 0.05). Participants in CBT reported greater reductions in overall anxiety levels (i.e., SCARED) from pre- (*M* = 35.50) to post-treatment (*M* = 28.75, *d* = 0.28), compared to the control (*M_pre_* = 33.35, *M_pos_*_t_ = 34.75, *d* = 0.07), *F*_1,7_ = 3.85, *p* < 0.05.	**Who:** graduate students **Setting:** classroom **Supervisor:** unclear ***Supervisor role:*** weekly face-to-face meeting and monitoring (e.g., role play upcoming session, discuss previous sessions, provide corrective feedback), and treatment notes **Duration:** 10 weekly sessions; 45 min each	Initial and ongoing training (didactic and interactive).
**Author(s) (Year):** Gray et al. (2024) **Study Design:** Quasi-experimental **Broader intervention:** Creating Opportunities for Personal Empowerment (COPE) for teens (brief, school-based CBT)	**Size:** *N* = 22 **Age Range:** 12–13 **Age M (SD):** 12.41 (0.49) **Gender:** 68.20% female, 31.82% male **Race/Ethnicity:** 22.70% Black or African American, 40.90% White, 22.70% Hispanic or Latino, 4.50% Asian/Pacific Islander, 9.10% other/mixed **Location:** Tennessee	Anxiety, depression	Thought identification, cognitive restructuring, problem-solving skills, psychoeducation, social skills, help-seeking skills, emotion identification, modeling, behavior analysis, mindfulness skills	No significant differences were observed across time for anxiety (i.e., GAD-7; *p* = 0.077) or depression (i.e., PHQ-A; *p* = 0.49); although, there was a decreasing trend from pre- (*M_anx_* = 9.00; *M_dep_* = 10.53) to post-intervention (*M_anx_* = 7.80; *M_dep_* = 9.47) to 2-month follow-up (*M_anx_*= 6.73; *M_dep_* = 8.80). Depression severity shifted from moderate to mild range by the 2-month follow-up, while anxiety severity remained in the mild range. Participants also reported decreased levels of functional impairment from anxiety and depression symptoms at the follow-up. Greater improvements were seen in participants with higher baseline symptoms: depression decreased by 3.5 points from pre-intervention to follow-up (severe to moderate range; *d* = 0.54, *p* = 0.12); anxiety significantly decreased by 4 points (mild to moderate range; *d* = 0.91, *p* = 0.02). Recent suicidal ideation endorsement increased from pre- (*n* = 5) to post-intervention (*n* = 8) then decreased at the follow-up below baseline (*n* = 2).	**Who:** nurses (RNs) **Setting:** classroom **Supervisor:** N/A ***Supervisor role:*** N/A **Duration:** 7 weekly sessions; 50–55 min each	Initial training (2–3 h).
**Author(s) (Year):** Heitkemper et al. (1993) **Study Design:** RCT **Broader intervention:** N/A	**Size:** *N* = 45 **Age Range:** 8–11 **Age M (SD):** N/A **Gender:** N/A **Race/Ethnicity:** N/A **Location:** Ohio	Anxiety (dental)	Relaxation skills	Paced respiration significantly lowered anxiety *(M_pre_* = 35.80, *M_post_* = 28.80) and expected discomfort (*M_pre_* = 5.00, *M_post_* = 2.10) more than cognitive coping (*p* < 0.05) and placebo (*p* < 0.05). Cognitive coping significantly reduced expected discomfort (*M_pre_* = 4.3, *M_post_* = 2.8) more than placebo (*p* < 0.05), but experiences of pain were not affected by either.	**Who:** audio recording played by dental assistant **Setting:** dentist’s office **Supervisor:** N/A ***Supervisor role:*** N/A **Duration:** 1 audiotape	N/A
**Author(s) (Year):** Hilt et al. (2024) **Study Design:** RCT **Broader intervention:** Brief, app-based mindfulness intervention	**Size:** *N* = 152 **Age Range:** 12–15 **Age M (SD):** 13.71 (0.89) **Gender:** 58.55% female, 41.45% male **Race/Ethnicity:** 82.24% White; 10.53% multiracial; 3.29% Black or African American; 1.97% Asian or Asian American; 1.32% other; 0.66% Native American or Pacific Islander; 10.53% Hispanic **Location:** Midwest of U.S.	Depression	Mindfulness skills	In a parallel mediation model, the mindfulness condition increased nonjudgment, which significantly reduced rumination at 6 weeks (*ab* = −1.32); no direct effect of condition on rumination. Only nonjudgment significantly increased in the mindfulness group (*p* < 0.05); marginal effects on awareness and describe. While nonreactivity also predicted lower rumination (*p* < 0.05), it was not affected by condition. A serial mediation pathway showed the mindfulness condition significantly reduced depression at 12 weeks (*p* < 0.01) and 6 months (*p* < 0.01) via increased nonjudgment (*p* < 0.05) and reduced rumination (*p* < 0.01). Nonjudgment significantly increased from pre- (*M* = 25.56) to post-treatment (*M* = 27.79) to 6 weeks (*M* = 28.09) and 6 months follow-up (*M* = 28.30), with a slight dip at 12 weeks (*M* = 27.46). Rumination significantly decreased from baseline (*M* = 17.81) to 6 weeks follow-up (*M* = 13.32), followed by slight increases at subsequent follow-ups (*M* = 14.60; 14.25).	**Who:** youth self-administered through an app **Setting:** internet **Supervisor:** N/A ***Supervisor role:*** N/A **Duration:** 3 daily exercises over 3 weeks; 3–12 min each	Initial training (i.e., orientation to app, practice).
**Author(s) (Year):** Jennings and Jennings (2013) **Study Design:** Quasi-experimental pilot **Broader intervention:** Brief mindfulness meditation training	**Size:** *N* = 8 **Age Range:** 17–18 **Age M (SD):** N/A **Gender:** 37.50% female, 62.50% male **Race/Ethnicity:** N/A **Location:** Pennsylvania	Anxiety	Relaxation skills, mindfulness skills	There was a 30% reduction in general anxiety scores (i.e., BAI) from pre- (*M* = 16.75, *SD* = 12.50) to post-mindfulness training (*M* = 12.00, *SD* = 5.60). More specifically, there was a 55% reduction in cognitive anxiety from pre- (*M* = 6.60, *SD* = 4.40) to post-training (*M* = 3.00, *SD* = 1.50), and an 11% reduction in physiological anxiety from pre- (*M* = 10.10, *SD* = 8.30) to post-training (*M* = 9.00, *SD* = 4.60). There was a 9% reduction in the ratings of social anxiety (i.e., IAS) from pre-(*M* = 38.90, *SD* = 9.60) to post-training (*M* = 35.50, *SD* = 8.20). The most salient areas of improvement pre- and post-mindfulness training were comfortability in a new group, fear the worst will happen, nervousness, tension around talking to new people, anxiety for parties, and fear of losing control. Notably, the three most salient areas on the BAI were from the cognitive subscale.	**Who:** peers (with no prior mindfulness experience) **Setting:** classroom **Supervisor:** experienced adult meditator ***Supervisor role:*** provide initial training **Duration:** 4 sessions within 3 weeks; 50 min each	Initial training (several hours).
**Author(s) (Year):** Joyce et al. (2010) **Study Design:** Quasi-experimental pilot **Broader intervention:** Mindfulness meditation program	**Size:** *N* = 141 **Age Range:** 10–13 **Age M (SD):** 11.40 (N/A) **Gender:** 43.80% female, 56.30% male **Race/Ethnicity:** N/A **Location:** Australia	Depression	Mindfulness skills	Participants scoring in borderline (b) and abnormal (a) severity for depression decreased from pre- (17.20% b; 8.60% a) to post-program (14.70% b, 6.90% a), *χ^2^*(4) = 59.95, *p* < 0.001. Depression scores significantly decreased for all participants from pre- (*M* = 9.28, *SD* = 7.55) to post-program (*M* = 7.86, *SD* = 6.53; *t*(119) = 2.96, *p* < 0.01, *d* = 0.27), with stronger improvements in those with higher baseline severity (M_pre_ = 19.80, M_post_ = 15.30; *t*(29) = 3.39, *p* < 0.01, *d* = 0.62). Teachers were generally positive about the program, noting students were more relaxed, focused, reflective, and open. Key facilitators included administrative, parent, and colleague support, classroom environment adjustments, student enthusiasm, and personal relevance of skills. Barriers included limited time, clearer manual integration of activities and lessons, and more professional development.	**Who:** teachers **Setting:** classroom **Supervisor:** course designer ***Supervisor role:*** provide initial training **Duration:** 10 lessons that could be flexibly delivered over 10 weeks; 45 min each	Initial training.
**Author(s) (Year):** Kalafat and Elias (1994) **Study Design:** Quasi-experimental **Broader intervention:** school-based suicide awareness curriculum	**Size:** *N* = 253 **Age Range:** N/A **Age M (SD):** N/A **Gender:** 43.00% female, 57.00% male **Race/Ethnicity:** N/A **Location:** Northeast of the U.S.	Suicidality	Psychoeducation, help-seeking skills	Participants in the suicide curriculum showed significantly greater suicide knowledge (*F*_1,233_ = 51.10, *p* < 0.0001), adaptive help-seeking attitudes (*F*_14,225_ = 1.87, *p* < 0.03), and intention to intervene with suicidal peers (e.g., tell trusted adult, recommend hotline or mental health center; *p* < 0.05). Many students rated the suicide classes as more interesting and helpful than other health classes, with most reporting that the curriculum would help them support peers.	**Who:** teachers (with general familiarity with suicidality) **Setting:** classroom **Supervisor:** curriculum authors ***Supervisor role:*** provide initial training **Duration:** 3 classes; 40–45 min each	Initial training (2.5 h).
**Author(s) (Year):** Kresovich et al. (2023) **Study Design:** Quasi-experimental **Broader intervention:** Smart Brain Wise Heart social-emotional learning intervention	**Size:** *N* = 462 **Age Range:** 13–16 **Age M (SD):** N/A **Gender:** 60.40% female, 39.60% male, 0% other gender identity **Race/Ethnicity:** Ninth grade class around 74.00% Hispanic or Latinx **Location:** Texas	Subjective wellbeing, depression, anxiety, suicidality	Thought identification, relaxation skills, psychoeducation, social skills, emotion identification, mindfulness skills	The intervention showed no significant main effects on resilience (*p* = 0.21), self-compassion (*p* = 0.17), hostility (*p* = 0.85), suicidal ideation (*p* = 0.40), peer violence victimization (*p* = 0.22), peer violence perpetration (*p* = 0.46), depression (*p* = 0.63), anxiety (*p* = 0.76), or hyperactivity (*p* = 0.51). However, for participants with lower academic achievement, the intervention group showed significantly improved resilience (*p* = 0.005), self-compassion (*p* < 0.05), and depression (*p* < 0.05) than the comparison group.	**Who:** teachers with web-based support **Setting:** classroom, internet **Supervisor:** intervention instructors ***Supervisor role:*** provide initial and ongoing training and support **Duration:** 8 weekly sessions; 45–55 min each	Initial training (voluntary, hybrid). Ongoing training (hybrid, individualized). On-demand support as needed.
**Author(s) (Year):** Melamed et al. (1978) **Study Design:** Experimental **Broader intervention:** N/A	**Size:** *N* = 80 **Age Range:** 4–11 **Age M (SD):** N/A **Gender:** N/A **Race/Ethnicity:** 27.50% White; 72.50% Black **Location:** Ohio	Anxiety (dental)	Modeling	Participants who viewed modeling videos reported fewer fears (F_1,56_ = 4.26, *p* < 0.04), were less disruptive (F_1,56_ = 7.06, *p* < 0.01), and had lower heart rate increases (F_1,51_ = 4.69, *p* < 0.035) compared to those who viewed demonstration videos. Modeling was especially effective for 6–8-year-olds, who were rated as less anxious (*t*(23) = 2.11, *p* < 0.05) and more cooperative by observers (*t*(23) = 2.30, *p* < 0.05) and dentists (*t*(23) = 3.30, *p* < 0.01). White children reported fewer fears after modeling videos (*t*(29) = 2.13, *p* < 0.05), whereas Black children reported fewer fears after demonstration videos (*t*(31) = 2.60, *p* < 0.02). 4–6-year-olds had fewer fears after longer model video (*t*(8) = 2.44, *p* < 0.05), although longer videos increased heart rate acceleration (*F*_4,212_ = 2.64, *p* < 0.04). Participants without prior experience were less disruptive after short model or long demonstration videos than short demonstration videos (*p* < 0.01); whereas those with experience were calmer after long model videos than long demonstration videos (*p* < 0.007). Self-reported dental fear predicted disruptive behavior (*r* = 0.29, *p* < 0.01), and younger participants showed greater anxiety.	**Who:** video-recording played by dental assistant **Setting:** dentist’s office **Supervisor:** N/A ***Supervisor role:*** N/A **Duration:** 1 video; 4–10 min	N/A
**Author(s) (Year):** Rudy et al. (2017) **Study Design:** RCT pilot **Broader intervention:** Parent-led exposure therapy	**Size:** *N* = 22 **Age Range:** 4–7 **Age M (SD):** 5.36 (1.14) **Gender:** 40.91% female, 59.09% male **Race/Ethnicity:** 63.64% White, 4.55% African American, 13.64% Hispanic, 4.55% Asian, 13.64% other or unreported **Location:** Florida	Anxiety	Exposure, psychoeducation, modeling	The exposure group showed significantly greater reduction in anxiety symptoms (i.e., ADIS-CSR, PARS, CSI-S) compared to treatment as usual group (*p* < 0.05). Among treatment completers, 90.91% of participants in exposure group were responders compared to 0% in treatment as usual group (*p* < 0.001). Anxiety diagnostic remission rates favored the exposure group (72.73%) over treatment as usual (11.11%; *p* = 0.01), which remained using intent-to-treat analyses (66.67% vs. 10.00%; *p* = 0.01).	**Who:** caregivers **Setting:** university-based clinic **Supervisor:** post-doctoral therapist ***Supervisor role:*** initial training, modeling, ongoing real-time feedback **Duration:** 10 sessions over 5 weeks (i.e., 2 times perweek); first session 90 min, all other sessions 60 min	Initial training. Ongoing training (i.e., modeled sessions, real-time coaching).
**Author(s) (Year):** Schibbye et al. (2024) **Study Design:** RCT **Broader intervention:** internet-based CBT (ICBT) for dental or injection phobia	**Size:** *N* = 33 **Age Range:** 8–15 **Age M (SD):** 11.20 (1.90) **Gender:** 63.64% female, 36.37% male **Race/Ethnicity:** N/A **Location:** Sweden	Anxiety (dental)	Cognitive restructuring, relaxation skills, exposure, psychoeducation, behavior analysis, mindfulness skills	Posttreatment, 41% of the ICBT group no longer met diagnostic criteria compared to 0% in the control group (*χ^2^* = 8.40, *p* = 0.004), and 65% of the ICBT group lost at least one phobia diagnosis compared to none in the control group (*χ^2^* = 15.50, *p* < 0.001). ICBT participants exhibited greater improvements in dental fear and anxiety (d = −1.60), negative cognitions (*d* = −1.30), injection fear (*d* = −1.00), self-efficacy (*d* = 2.00), and parent-reported fear and anxiety (*d* = −1.30); *p* < 0.01; and managed more dental procedures at follow-up (child: *p* < 0.001, *d* = 1.60; parent: *p* = 0.009, *d* = 1.00). There were no significant differences in parent self-efficacy (*p* = 0.07, *d* = 0.70).	**Who:** caregivers with web-support **Setting:** home, internet, dentist office **Supervisor:** therapist ***Supervisor role:*** ongoing answer questions and provide feedback **Duration:** 12 weekly modules	Initial training. Ongoing training (i.e., feedback on modules and practice, answer questions, monitor completion, reminders). On-demand support as needed.
**Author(s) (Year):** Swierad and Williams (2023) **Study Design:** Quasi-experimental pilot **Broader intervention:** CASEL framework web-based intervention	**Size:** *N* = 57 **Age Range:** 10–11 **Age M (SD):** N/A **Gender:** N/A **Race/Ethnicity:** N/A **Location:** New York	Subjective wellbeing	Mindfulness skills, gratitude, emotion identification	Students’ reactions to the program were positive, with key themes of user-friendliness, appreciating variety of activities, learning tools to help them cope with stress and stay calm, and recommending miscellaneous improvement (e.g., more videos). Most students expressed positive attitudes towards the mindfulness and gratitude modules (e.g., made them feel calm, fun), with 86% having a strong favorable response to the overall activities presented in modules and 14% disliking some activities in the modules. There were significant increases in life satisfaction (*F*_2,70_ = 3.39, *p* = 0.03) from post-intervention (*M* = 4.32) to 1-week follow-up (*M* = 4.54, *p* = 0.00); with follow-up being similar to baseline (*M* = 4.37, *p* = 0.12). Gratitude also significantly improved (*F*_2,74_ = 4.95, *p* = 0.01) from baseline (*M* = 11.71) to post-intervention (*M* = 12.78, *p* = 0.01) and to 1-week follow-up (*M* = 12.71, *p* = 0.02). There were no significant changes in mindfulness from pre- to post-intervention to follow-up, *F*_2,74_ = 0.35, *p* = 0.966).	**Who:** youth self-administered through online game **Setting:** classroom, internet **Supervisor:** teachers ***Supervisor role:*** oversee self-administration **Duration:** self-administered game modules and reflection session; 40–45 min total	N/A
**Author(s) (Year):** Tol et al. (2008) **Study Design:** Cluster randomized trial **Broader intervention:** school-based group mental health intervention	**Size:** *N* = 495 **Age Range:** 7–15 **Age M (SD):** 9.90 (1.30) **Gender:** 48.60% female, 51.40% male **Race/Ethnicity:** N/A **Location:** Indonesia	Anxiety, depression, subjective wellbeing	Cognitive restructuring, relaxation skills, psychoeducation, activity scheduling, trauma narrative, mindfulness skills	Participants in the intervention showed significantly greater improvements from baseline to post-intervention than the control on all child-rated outcomes except anxiety (*p* = 0.15, *d* = 0.14) including PTSD symptoms (*p* < 0.001, *d* = 0.55), traumatic stress (*p* = 0.03, *d* = 0.21), depressive symptoms (*p* = 0.002, *d* = 0.31), functional impairment (*p* < 0.001, *d* = 0.42), and hope (*p* = 0.004, *d* = 0.29). No significant changes were seen in scores between post-intervention and 6-month follow-up, suggesting the effects remained with smaller magnitude, and hope showed moderate increase in effect size over time (*d* = 0.04, 0.44, 0.21, 0.24, 0.26, 0.38; respectively). Mixed-methods regression confirmed significant treatment effects on PTSD symptoms (*M_difference_* = −2.78; 95% CI [1.02, 4.53]) and hope (*M_differenc_*_e_ = 2.21, 95% CI [−3.52, −0.91]), but no other outcomes. Sex moderated effects, with girls showing greater improvement in PTSD (*B* = 4.76; 95% CI [2.49, 7.03]), functional impairment (*B* = 1.64; 95% CI [0.27, 3.02]) and hope (*B* = −2.71; 95% CI [−4.61, −0.82]), while boys showed no significant effects.	**Who:** unspecified local paraprofessionals **Setting:** classroom **Supervisor:** independent research assistants * ***Supervisor role:*** fidelity checks *[* note: supervisors completed 5-week training course]* **Duration:** 15 sessions over 5 weeks (i.e., 3 times perweek)	Initial training (2 weeks; unclear who delivers it). Fidelity checks from videotaped sessions.
**Author(s) (Year):** Volungis (2020) **Study Design:** Quasi-experimental pilot **Broader intervention:** Signs of Suicide (SOS) prevention program	**Size:** *N* = 2130 **Age Range:** 13–18 **Age M * (SD):** 15.5 (N/A) **Gender *:** 52.00% female, 48.00% male **Race/Ethnicity *:** 90% Caucasian **Location:** New England * *Note: based on school data rather than specific sample.*	Depression, suicidality	Psychoeducation, social skills, help-seeking skills	Across all three years, the results from the pre-survey (Y1: *M* = 21.40, *SD* = 4.03; Y2: *M* = 33.00, *SD* = 7.68; Y3: *M* = 28.85, *SD* = 6.60) and post-survey (Y1: *M* = 25.86, *SD* = 2.57; Y2: *M* = 43.80, *SD* = 7.94; Y3: *M* = 33.25, *SD* = 6.70) indicate that the SOS prevention program improved students’ knowledge and awareness of depression and suicide, including receiving help for themselves and their peers (Y1: *t*(816) = 32.84, *p* < 0.001, *d* = 1.32; Y2: *t*(673) = 30.90, *p*< 0.001, *d* = 1.38; Y3: *t*(398) = 11.481, *p* < 0.001, *d* = 0.65).	**Who:** teachers **Setting:** classroom **Supervisor:** school counselors * ***Supervisor role:*** initial training, ongoing monthly check-ins, available for ongoing support as needed *[* note: supervisors were trained for 3 h by a Sandy Hook Promise trainer]* **Duration:** 2-day program once every year over 3 years; 75 min total each year	Information given to review prior to training. Initial and ongoing training.
**Author(s) (Year):** Yeo et al. (2016) **Study Design:** Quasi-experimental **Broader intervention:** classroom-based CBT test anxiety prevention program	**Size:** *N* = 115 **Age Range:** 9–12 **Age M (SD):** 10.15 (0.50) **Gender:** 39.13% female, 60.87% male **Race/Ethnicity:** 68.70% Chinese, 9.57% Malay, 7.83% Indian, 13.91% other **Location:** Singapore	Anxiety (exams)	Relaxation skills, study skills, mindfulness skills	Students in the CBT group (M_difference_ = 0.26) showed significant reductions in test anxiety 2 months post-intervention compared those in the control (M_difference_ = −0.01); *t*(113) = −2.74, *p* = 0.007, *d* = 0.52. In the CBT group, baseline anxiety levels moderated decreases in test anxiety– those with high baseline severity showed significant differences at post-intervention and follow-up (*F*_2,51_ = 10.14, *p* < 0.0001, *η*^2^ = 0.29). In the control group, there were significant differences in test anxiety scores across achievement levels (*F*_2,54_ = 4.5, *p* < 0.05)—average achieving students reported increasing test anxiety from baseline (*M* = 2.62) to post-intervention (*M* = 2.66,) to follow-up (*M* = 2.87), whereas their high and low achieving peers were fairly consistent in their anxiety levels over time. In the CBT group, students showed reduced anxiety over time regardless of achievement level (*F*_2,54_ = 5.33, *p* < 0.01). 6 of the 7 CBT skills were effective for reducing anxiety (i.e., balloon breathing, following own time-table at home, muscle relaxation, thinking of a special memory to feel calm and good, paying attention to body feelings, practicing breathing and saying positive things to self simultaneously), while positive self-talk was not.	**Who:** graduate students **Setting:** classroom **Supervisor:** N/A ***Supervisor role:*** N/A **Duration:** 4 weekly sessions; 30 min each	N/A

**Table A2:** Synthesis of Evidence-Based Ingredients.

Ingredient (# of Studies)	Description	Examples (Studies)	Measured Outcomes
Mindfulness skills (11)	Activities to practice being fully present and aware of your current experiences (e.g., thoughts, body sensations, environment) without judgement.	Focusing on a body sensations (Yeo et al., 2016), meditation focused on breath (Hilt et al., 2024), imagining a peaceful space (Jennings & Jennings, 2013), focusing on an object in nature (Jennings & Jennings, 2013), accepting disruptions while focusing (Jennings & Jennings, 2013), nonjudgemental awareness (Hilt et al., 2024), listening to song and focusing on rhythm, lyrics, instruments, and/or volume (Swierad & Williams, 2023)	Anxiety, depression, suicidality, subjective wellbeing
Relaxation skills (9)	Activities aimed to decrease tension and stress on the mind and body.	Balloon breathing (Yeo et al., 2016), heart-focused breathing (Kresovich et al., 2023), paced respiration (Heitkemper et al., 1993), progressive muscle relaxation, diaphragmatic breathing (Ginsburg & Drake, 2002), belly breathing (Swierad & Williams, 2023)	Anxiety, depression, suicidality, subjective wellbeing
Psychoeducation (9)	Providing knowledge and information about a specific topic to better understand it.	Prevalence of depression and suicide, myths, risk and protective factors, warning signs, community resources (Volungis, 2020), function of amygdala and thalamus (Kresovich), cognitive-behavioral therapy rationale (Ginsburg & Drake, 2002)	Anxiety, depression, suicidality, subjective wellbeing
Exposure (6)	Introducing an individual to feared or challenging stimuli, sometimes in a gradual fashion (e.g., fear ladder, setting small goals) and sometimes with rewards for completing feared behavior.	Completing exposures of increasing difficulty (Bilek et al., 2022), in vivo and out-of-session gradual exposures to hierarchical feared stimuli with self-reward systems (Ginsburg & Drake, 2002), watching recordings of feared dental procedures, visiting feared dentist office (Schibbye et al., 2024)	Anxiety
Cognitive restructuring (6)	Reframing and/or changing thought patterns.	Practicing positive thinking and self-talk, stopping negative thoughts (Gray et al., 2024), cognitive restructuring (Ginsburg & Drake, 2002), learning to swap negative thoughts for helpful ones or SPARX—smart, positive, active, realistic, X-factor thoughts (Fleming et al., 2016)	Anxiety, depression, subjective wellbeing
Social or interpersonal skills (5)	Activities aimed at fostering skills for youth to socially interact with one another.	Developing communication skills (Kresovich et al., 2023), identifying how to effectively communicate thoughts (Gray et al., 2024), assertiveness, listening, and negotiation skills (Fleming et al., 2016)	Anxiety, depression, suicidality, subjective wellbeing
Thought identification (4)	Recognizing thoughts and identifying thought patterns.	Identifying negative thoughts (Gray et al., 2024), identifying ‘reactive’ brain vs. ‘thinking’ brain (Kresovich et al., 2023), identifying and challenging anxious thoughts (P. M. Barrett et al., 2001), recognizing GNATS or gloomy negative automatic thoughts (Fleming et al., 2016)	Anxiety, depression, suicidality, subjective wellbeing
Help-seeking skills (3)	Recognizing an individual or situation that needs assistance, and requesting support from others.	Identifying warning signs of depression and suicide and learning how to seek help (e.g., connect with community resources) for themselves or a peer (Volungis, 2020), identifying suicidal peers and learning how to request help from an adult (Kalafat & Elias, 1994)	Anxiety, depression, suicidality
Emotion identification (3)	Recognizing and understanding one’s own emotions.	Identifying how emotions impact their behavior and decisions, identifying when they feel ‘in sync’ and’ out of sync’ with emotions (Kresovich et al., 2023), naming their emotions (Swierad & Williams, 2023)	Anxiety, depression, suicidality, subjective wellbeing
Problem-solving skills (3)	Identifying a problem, analyzing it, and finding effective solutions.	Creating a plan to use relaxation techniques to reset after stressful situations (Kresovich et al., 2023), identifying anxiety-provoking situations and creating a problem-solving plan for coping (P. M. Barrett et al., 2001), problem solving and setting goals (Gray et al., 2024), STEPS: say the problem, think of solutions, examine the pros and cons, pick one and try it, see what happens (Fleming et al., 2016)	Anxiety, depression
Activity scheduling (2)	Intentionally planning and engaging in activities that bring pleasure, despite one’s motivation.	Behavioral activation and scheduling activities (Fleming et al., 2016), reconnecting with social contexts through cooperative play and creative expression activities (Tol et al., 2008)	Anxiety, depression, subjective wellbeing
Modeling (2)	A technique where an individual demonstrates a specific behavior to another individual.	Watch film of peer completing feared dental procedure (Melamed et al., 1978), watch an individual complete feared behavior (Rudy et al., 2017)	Anxiety
Behavior analysis (2)	Acknowledging and understanding how different factors (e.g., environmental, individual, interpersonal, systemic) impact one’s behavior.	Understanding how their thoughts and feelings relate to their behavior (Gray et al., 2024), analyzing behavior patterns (Schibbye et al., 2024)	Anxiety, depression
Expressions of hope (1)	Positive action-oriented thoughts and/or expressions about the future.	Explanations that people recover from depression, statements that provide a sense of hope (Fleming et al., 2016)	Depression
Gratitude (1)	Recognizing and appreciating positive things in life.	List out the things they are thankful for in the present moment, gratitude jar, write a thank you note to a friend, and make the background of their phone something they are thankful for (Swierad & Williams, 2023)	Subjective wellbeing
Trauma narrative (1)	Creating and processing a story of a traumatic event that includes the individual’s feelings, thoughts, and experiences.	Drafting a trauma narrative (Tol et al., 2008)	Anxiety, depression, subjective wellbeing
Study skills (1)	Strategies or activities that help students achieve academically, which can range from organization and memorization techniques to practicing specific problems to increase competency.	Completing timetables at home (Yeo et al., 2016)	Anxiety
